# Interdisciplinary Approaches to Deal with Alzheimer’s Disease—From Bench to Bedside: What Feasible Options Do Already Exist Today?

**DOI:** 10.3390/biomedicines10112922

**Published:** 2022-11-14

**Authors:** Irene Ablinger, Katharina Dressel, Thea Rott, Anna Andrea Lauer, Michael Tiemann, João Pedro Batista, Tim Taddey, Heike Sabine Grimm, Marcus Otto Walter Grimm

**Affiliations:** 1 Speech and Language Therapy, Campus Bonn, SRH University of Applied Health Sciences, 53111 Bonn, Germany; 2 Speech and Language Therapy, Campus Düsseldorf, SRH University of Applied Health Sciences, 40210 Düsseldorf, Germany; 3 Interdisciplinary Periodontology and Prevention, Campus Rheinland, SRH University of Applied Health Sciences, 51377 Leverkusen, Germany; 4 Nutrition Therapy and Counseling, Campus Rheinland, SRH University of Applied Health Sciences, 51377 Leverkusen, Germany; 5 Experimental Neurology, Saarland University, 66424 Homburg, Germany; 6 Sport Science, Campus Rheinland, SRH University of Applied Health Sciences, 51377 Leverkusen, Germany; 7 Sport Science and Physiotherapy, Campus Rheinland, SRH University of Applied Health Sciences, 51377 Leverkusen, Germany; 8 Physiotherapy, Campus Rheinland, SRH University of Applied Health Sciences, 51377 Leverkusen, Germany

**Keywords:** Alzheimer’s disease, nutritional approaches, physical activity, socioeconomic factors, cognition-oriented treatment, communication, oral health, interdisciplinary approaches, multimodal intervention, multidomain intervention

## Abstract

Alzheimer’s disease is one of the most common neurodegenerative diseases in the western population. The incidence of this disease increases with age. Rising life expectancy and the resulting increase in the ratio of elderly in the population are likely to exacerbate socioeconomic problems. Alzheimer’s disease is a multifactorial disease. In addition to amyloidogenic processing leading to plaques, and tau pathology, but also other molecular causes such as oxidative stress or inflammation play a crucial role. We summarize the molecular mechanisms leading to Alzheimer’s disease and which potential interventions are known to interfere with these mechanisms, focusing on nutritional approaches and physical activity but also the beneficial effects of cognition-oriented treatments with a focus on language and communication. Interestingly, recent findings also suggest a causal link between oral conditions, such as periodontitis or edentulism, and Alzheimer’s disease, raising the question of whether dental intervention in Alzheimer’s patients can be beneficial as well. Unfortunately, all previous single-domain interventions have been shown to have limited benefit to patients. However, the latest studies indicate that combining these efforts into multidomain approaches may have increased preventive or therapeutic potential. Therefore, as another emphasis in this review, we provide an overview of current literature dealing with studies combining the above-mentioned approaches and discuss potential advantages compared to monotherapies. Considering current literature and intervention options, we also propose a multidomain interdisciplinary approach for the treatment of Alzheimer’s disease patients that synergistically links the individual approaches. In conclusion, this review highlights the need to combine different approaches in an interdisciplinary manner, to address the future challenges of Alzheimer’s disease.

## 1. Introduction

Alzheimer’s disease (AD) is the most prevalent form of dementia and is characterized by behavioral and cognitive impairments. It results in a loss of memory and acquired skills, as well as a decrease in participation in daily activities, communication skills, social interactions and quality of life leading to an increasing burden on caregivers [1,2]. The main histopathological hallmarks of AD are the severe accumulation of amyloid-β in extracellular neuritic plaques as well as intracellular neurofibrillary tangles (NFTs) in vulnerable brain regions such as the hippocampus and cortex [3]. Based on the onset of symptoms and pathological changes in the cortex and hippocampus, clinical AD stages can be classified into at least four phases. The pre-symptomatic/pre-clinical stage is asymptomatic despite early pathological changes in the cortex and hippocampal formation [4,5,6], followed by mild cognitive impairment (MCI). MCI shows limitations in some cognitive domains on functional examination without restrictions in the ability to cope with everyday life [7,8,9] but definite laboratory evidence, including biomarkers such as low amyloid-β and increased tau proteins in the cerebrospinal fluid [5]. Mild/early dementia due to AD is characterized by symptoms such as spatial and temporal disorientation, loss of memory and concentration, word-finding difficulties, and the development of depression. In the moderate stage of AD, increased memory loss, impairment of visuospatial abilities leading to difficulties in recognizing family members and friends, and problems with language and communication occur [5,6,10]. The final stage of AD, severe/late AD, is associated with severe accumulation of NTFs and senile plaques in the entire cortex area, resulting in advanced functional and cognitive impairment, incontinence, dysphagia, and complete dependence on caregivers [5].

Currently (2020), AD is estimated to affect 55 million people worldwide. Global AD prevalence is predicted to increase to 139 million people living with AD by 2050 due to the aging population, making AD a major public health concern. Yet, to date, there are only two classes of approved drugs to treat persons with Alzheimer’s disease (PwAD): Cholinesterase enzyme inhibitors and N-methyl-D-aspartate (NMDA) inhibitors. Acetylcholine-producing cells are destroyed in AD by different physiological processes. Treatment with acetylcholinesterase inhibitors such as donepezil, galantamine and rivastigmine blocks the catabolism of acetylcholine, increasing acetylcholine concentration in the synaptic cleft and thus cholinergic transmission in the brain [5,11,12,13]. Overactivation of NMDA-receptors leads to increased intracellular calcium levels, promoting cell death and synaptic dysfunction. The use of partial NMDA antagonists such as memantine, which can be taken in combination with cholinesterase inhibitors, prevents NMDA-receptor overactivation and restores its normal activity [14,15]. However, these medications are only temporary effective in treating the symptoms of AD by improving quality of life but do not cure or prevent the disease.

Most AD cases occur sporadically (known as sporadic Alzheimer’s disease, SAD) with the age of onset above 65 years. Less than 10% of AD cases are caused by genetic familial mutations leading to earlier disease onset, usually between the age of 30 and 60 (known as early onset Alzheimer’s disease, EOAD or familial Alzheimer’s disease, FAD). AD is considered a multifactorial disease as it is characterized by impairments in multiple cellular processes. In addition to cholinergic dysfunction and the well-known Abeta (Aβ) and Tau pathology of AD, inflammation, oxidative stress, as well as, e.g., alterations in lipid and energy metabolism are also involved in the pathogenesis of AD.

The Lancet Commission on dementia prevention, intervention and care [16] recently identified 12 potentially modifiable risk factors for dementia that account for around 40% of dementia cases worldwide. These include, among others, physical inactivity, low social contact, obesity, and associated diabetes. Frequent physical activity, promotion of communication to maintain social interactions, and a healthy diet to avoid the risks of obesity and diabetes may therefore affect neuropathological damage and cognitive reserve [16] and contribute to the prevention or delay of dementia. According to current literature, the intraoral condition could also represent such a modifiable risk factor [17,18,19].

Reducing modifiable risk factors, in addition to pharmacological interventions, is an important approach in dementia treatment. According to Spector and Orrell’s biopsychosocial (BPS) model [20], dementia is a multifactorial disease in which psychosocial and biological processes are interrelated. Both domains include fixed, non-modifiable factors (e.g., age; education) and tractable, modifiable factors (e.g., physical health; social interaction, mental stimulation). Treatment plans should be tailored to the individual’s needs. Factors amenable to change are identified with the aim of influencing them through medical and non-pharmacological interventions [20,21]. In recent years, non-pharmacological interventions have become increasingly important in the management of dementia and in the effort to improve living with dementia and maintain quality of life [1,7,22,23,24]. Non-pharmacological interventions include evidence-based psychological, bodily, nutritional, digital or basic methods and approaches, that are individually selected and adjusted to the persons’ needs in their courses of disease [7,24]. Among these, cognition-oriented treatments (COT) [1,24,25], physical activity [26], and diet [27] form an integral part.

### 1.1. Aim of This Review

To date, there is no pharmaceutical or antibody-based causal therapy for the treatment of AD. As demographics change, alternative treatments for this devasting neurodegenerative disease will be needed. In this review, we summarize different nutritional approaches and their underlying mechanisms, the effect of physical activity, cognition-oriented treatments with a focus on communication, and the potential advantages of socioenvironmental factors in relation to AD. In addition, the current literature suggests that oral status, particularly periodontitis and edentulism, are also mechanistically linked to AD. This represents another interesting new approach in the treatment of AD. However, any of the aforementioned treatment approaches applied individually show limited benefits in treating PwAD. Therefore, recent efforts are focused on combining different approaches as multidisciplinary interventions. In the second part of our review, we summarize the results of the first multidomain interventions. Finally, we propose a multidisciplinary, individualized treatment, based on an even stronger integration of the different disciplines and already existing treatment methods.

### 1.2. Molecular Mechanisms Involved in AD Pathogenesis

The amyloid pathology of AD is caused by sequential proteolytic cleavage of the amyloid precursor protein (APP), resulting in Aβ monomers, that aggregate to oligomeric Aβ, discussed as the most neurotoxic form, and finally to Aβ fibrils and plaques [28,29]. The type-I transmembrane protein APP belongs to an evolutionarily conserved protein family and is ubiquitously expressed in mammals [30,31]. However, the function of APP is still debated, with the discussion that it plays a role in cell health, growth, gene transcription and lipid homeostasis [32]. The release of Aβ peptides out of APP is a physiological process, that occurs throughout life and depends on whether APP is primarily processed in the non-amyloidogenic or amyloidogenic pathway. The non-amyloidogenic pathway prevents the release of Aβ peptides out of APP and thus the formation of Aβ plaques. The initial ectodomain shedding of APP in the non-amyloidogenic pathway is realized by α-secretases, cleaving APP within the Aβ domain. The activity of the identified α-secretases ADAM9, ADAM10 and ADAM17 [33,34], belonging to the ADAM family (a disintegrin and metalloprotease), generates soluble α-secreted APP (sAPPα) and the membrane-tethered C-terminal fragment αCTF, further processed by γ-secretase, leading to the non-toxic peptide p3. Furthermore, sAPPα has neuroprotective and memory-enhancing effects [35,36,37]. In the amyloidogenic pathway, APP is first cleaved by β-secretase, generating the N-terminus of Aβ. The membrane-bound aspartyl protease BACE 1 (β-site APP cleaving enzyme) has been identified as the main β-secretase [38], mainly found in intracellular compartments with an acidic pH such as late Golgi-compartments and endosomes [39,40]. Similar to α-secretase cleavage of APP, BACE-cleavage leads to the release of a soluble fragment, β-secreted APP (sAPPβ), and a C-terminal membrane-embedded fragment called βCTF [39,41]. Subsequent cleavage of βCTF by γ-secretase within the transmembrane domain results in the generation of Aβ peptides varying in their length at the C-terminus [42,43]. Aβ40 peptides have been found to be the most abundant Aβ peptides (80–90% of Aβ peptides) whereas the more hydrophobic Aβ42 peptides represent only approximately 10% of Aβ peptides. The heterotetrameric protein complex leading to γ-secretase activity includes the transmembrane proteins presenilin1 (PS1) or presenilin 2 (PS2), identified as the catalytically active components, anterior pharynx defective 1 (APH-1), presenilin enhancer 2 (PEN-2) and nicastrin (NCT) [44,45]. All components of the γ-secretase complex as well as β-secretase BACE1 have been found in lipid rafts, small membrane microdomains enriched in sphingolipids and cholesterol [46,47]. Vice versa, non-amyloidogenic α-secretase processing of APP was reported to be localized in non-raft membrane domains, indicating that APP processing is highly dependent on membrane lipid composition.

In addition to amyloid plaques, neurofibrillary tangles (NFTs) are considered a key pathological feature of AD. Intraneuronal NFTs are composed of hyperphosphorylated tau proteins aggregated as insoluble paired helical fragments inside neurons [48,49,50]. Tau proteins that can bind to tubulin monomers are mainly expressed in neurons [51,52] and are important to stabilize the neuronal microtubule network, being essential for maintaining cell shape and transport along axons. Tau activity and tau function are regulated by phosphorylation, involving protein kinases and protein phosphatases [53,54]. Activation of kinases and/or a decrease in the activity of phosphatases are discussed to result in Tau hyperphosphorylation in AD. Several kinases involved in Tau hyperphosphorylation and tangle-like filament morphology have been identified, including, e.g., glycogen synthase kinase-3β (GSK3β), protein kinase A (PKA), the calcium and calmodulin-dependent protein kinase-II (CaMKII) as well as cyclin-dependent kinase 5 (CDK5) [55]. Furthermore, mitogen-activated protein (MAP) kinases have been reported to result in tau hyperphosphorylation, e.g., ERK1 and ERK2, p38, c-Jun N-terminal kinase (JNK) and p70S6 kinase [55]. Tau dephosphorylation has been found to be catalyzed by the protein phosphatases PP1, PP2A, PP2B and PP5 in vitro and in vivo [56]. Among these phosphatases, PP2A is discussed as one of the main enzymes preventing hyperphosphorylation of tau in AD [57,58]. Interestingly, the activity of the tau kinases ERK1 and ERK2, PKA, CaMKII and p70S6 is regulated by PP2A, which has been found to be reduced in AD brains, emphasizing the role of PP2A in tau hyperphosphorylation in AD.

In addition to the Aβ and tau pathology, the occurrence of a continuous immune response in the brain is considered a third core pathology of AD [59]. Acute inflammation by activated microglia and other immune cells in the brain serves to defend against toxins, brain injury and infections and plays a neuroprotective role during the acute-phase response. In early AD pathogenesis, the enhanced immune response leads to the clearance of Aβ by activated microglia and has been shown to have beneficial effects on AD-related pathologies in several animal models [60,61]. When there is an imbalance between pro-inflammatory and anti-inflammatory signaling, as reported in AD, chronic neuroinflammation occurs [62,63], leading to the release of pro-inflammatory and toxic products, including reactive oxidative species (ROS), nitric oxide and cytokines, e.g., interleukin-1 (IL-1), interleukin 1β (IL-1β), and tumor necrosis factor-α (TNFα). Sustained activation of microglia has been shown to exacerbate both Aβ and Tau pathology, thus linking neuroinflammation to the other two core pathologies. For example, IL-1 has been reported to be responsible for elevated APP production and Aβ load [64], whereas interleukin-1β increases levels of interleukin-6, which is known to stimulate CDK5, a well-known kinase involved in Tau hyperphosphorylation [65].

Similar to the imbalance between pro-inflammatory and anti-inflammatory signaling, the imbalance between the generation and detoxification of ROS leading to oxidative stress is also closely linked to the pathogenesis of AD [66]. This delicate balance between the rate of ROS generation and ROS clearance is warranted by antioxidants and related enzymes. Elevated levels of ROS, resulting from either increased ROS production or impaired antioxidative system, lead to oxidative damage of different biomolecules, including lipid peroxidation, protein oxidation and oxidation of nucleic acids. The important role of oxidative stress in AD is also supported by the findings that oxidative imbalance is an early event in AD pathogenesis. In individuals suffering from MCI, significant oxidative imbalance, increased total protein peroxidation, and oxidative modification of specific proteins have been found in the hippocampus and superior and middle temporal gyri [67,68,69]. Furthermore, significantly decreased levels of non-enzymatic antioxidants, such as vitamin C, vitamin A, vitamin E, and lutein, as well as decreased levels of antioxidant enzymes, such as superoxide dismutase, glutathione peroxidase, and glutathione reductase were found in MCI [67,69]. Additionally, structurally, and functionally damaged mitochondria, which produce ROS more efficiently and ATP less efficiently, were found to be an early and prominent feature of AD [70,71].

## 2. Individual Approaches

### 2.1. Nutritional Approaches: Molecular Mechanisms of Dietary Fatty Acids and Vitamins in the Development of Alzheimer’s Disease

As mentioned above, diet is discussed as an AD risk factor. Dietary interventions to prevent or delay AD, including various vitamins, dietary fatty acids (FA) and herbal ingredients are mainly associated with Aβ and Tau pathology of AD and affect inflammation and oxidative stress, including mitochondrial damage [72,73,74,75].

Importantly, the brain is highly enriched in lipids, accounting for at least 50% of dry brain weight [76]. Lipids, as basic structural components of neuronal cell membranes, play an important role in human health and brain function. Disruption of lipid homeostasis is closely associated with neurological disorders and neurodegenerative diseases such as AD. Moreover, aging in general is associated with changes in cerebral lipid content and composition. For example, it has been shown, that ethanolamine plasmalogens are decreased until the age of 70 and sphingomyelin levels were reduced by around 20% until the age of 100 years. Additionally, a progressive decline in polyunsaturated fatty acids (PUFAs) during aging in particular in DHA has been reported. Importantly, a decrease in plasmalogens, PUFA and sphingomyelins has been additionally discussed to be associated with an increased risk of Alzheimer’s disease [76,77,78].

The brain is particularly rich in long-chain polyunsaturated fatty acids (PUFAs) docosahexaenoic acid (DHA; 22:6 n-3) and arachidonic acid (AA; 20:4 n-6), representing precursors for the biosynthesis of lipid mediators that control the inflammatory response. The omega-6 PUFAs such as AA represent precursors of pro-inflammatory eicosanoids, whereas the n-3 PUFA DHA exerts anti-inflammatory, antioxidant and neuroprotective effects [79]. Besides the involvement of lipid membrane composition in brain function and disease, vitamins are discussed to modulate AD progression due to their antioxidative properties and important role in the homocysteine/methionine cycle [80,81].

#### 2.1.1. Effect on Aβ Pathology

##### Non-Amyloidogenic α-Secretase Processing of APP

**Figure 1:**
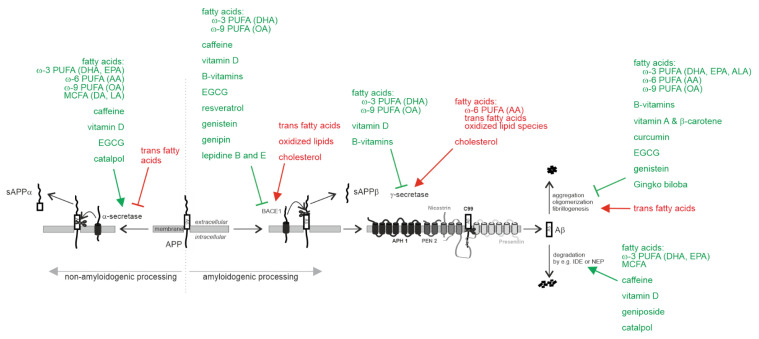
Influence of nutritional components on Aβ pathology. PUFA: polyunsaturated fatty acid. DHA: docosahexaenoic acid. EPA: eicosapentaenoic acid. AA: arachidonic acid. MCFA: medium-chain saturated fatty acids. OA: oleic acid. DA: decanoic acid. LA: lauric acid. EGCG: epigallocatechin gallate.

Dietary interventions modulating the Aβ-preventing *α*-secretase cleavage of APP are a promising target for the treatment of AD (Figure 1).

DHA is the most abundant n-3 PUFA in all brain regions [82]. It is discussed as one of the most important FAs for the treatment or prevention of AD. It was found that *α*-secretase processing of APP, which reduces Aβ formation is stimulated in DHA-treated cellular models of AD, including neuroblastoma cells overexpressing APP695wildtype or APP695 with the Swedish double mutation (K670N, M671L) or both the Swedish and the Arctic (E693G) mutations as well as APP695 transfected HEK cells [83,84,85]. The molecular mechanisms leading to increased *α*-secretase processing of APP in the presence of DHA are due to increased *ADAM17* gene expression and increased ADAM17 protein stability [84]. Notably, small amounts of oxidized DHA reversed the protective effect of DHA, decreasing the *α*-secretase processing of APP [86]. Besides DHA, both the n-3 PUFA eicosapentaenoic acid (EPA) and the n-6 PUFA AA increased *α*-secreted APP (sAPP*α*) in differentiated human neuroblastoma SH-SY5Y cells [87]. Increased levels of sAPP*α* have been also found in an early onset AD transgenic (Tg) mouse model fed with a low-fat, cholesterol-free diet enriched with the n-9 PUFA oleic acid (OA) [88]. The *α*-secretase stimulating effect is not limited to PUFAs, as phospholipids containing medium-chain saturated fatty acids (MCFAs)—decanoic acid (10:0) and lauric acid (12:0)—also increase *α*-secretase activity in human neuroblastoma cells [89]. In addition, calcifediol (25OH vitamin D3) has been shown to increase *α*-secretase activity promoting the non-amyloidogenic processing of APP [90]. In contrast, trans-fatty acids, whose consumption has increased in the 20th century since the first successful hydrogenation of oils [91], have been found to decrease non-amyloidogenic processing of APP by decreasing *ADAM10* gene expression, resulting in elevated production of Aβ [92]. This effect is mediated by a decrease in *ADAM10* gene expression and ADAM10 protein levels. Furthermore, high concentrations of trans fatty acids have been reported to decrease brain DHA in a 3xTg-AD mouse model of AD [93]. However, no significant effect on major brain neuropathological hallmarks of AD, including levels of Aβ40 and Aβ42 levels, was found in this study.

It has been reported that, in addition to FA and vitamins, dietary phytochemicals reduce the Aβ generation by increasing the α-secretase processing of APP. The polyphenol epigallocatechin-3-gallate (EGCG), a major catechin present in green tea, has been shown to decrease Aβ generation in neurons overexpressing APP695 and to decrease Aβ levels and plaques in Tg2576 AD mice, expressing the Swedish mutant of APP [94,95]. The EGCG-induced reduction in Aβ was attributed to an elevation in α-secretase APP cleavage, leading to an increase in α-CTF generation and sAPPα release, caused by a significant increase in the expression of ADAM10 and TNF-α converting enzyme, a further α-secretase candidate [94,96]. EGCG also significantly decreased brain Aβ production and plaque burden by increasing the levels of α-secretase ADAM10 in high-fat-fed transgenic APPswe/PSdE9 mice [97]. In vivo, intraperitoneal treatment of transgenic APPswedish Tg2576 mice with EGCG for 2 months significantly decreased Aβ levels [95]. Catalpol, an iridoid glycoside extracted from *Rehmannia glutinosa* roots, elevated the expression of α-secretase, promoting non-amyloidogenic APP processing in neuronal N2a cells overexpressing the Swedish mutant of APP [98]. An increase in protein stability of the α-secretase ADAM10 was identified as the molecular mechanism underlying the α-secretase stimulating effect in cell cultures, treated with methylxanthines, including caffeine, a natural alkaloid stimulant of coffee beans [99].

##### Amyloidogenic Processing of APP by β-Site Cleaving Enzyme BACE1 and γ-Secretase

Besides the beneficial property of DHA to increase the α-secretase processing of APP, DHA was found to decrease the amyloidogenic processing of APP by β- and γ-secretase [84] (Figure 1). In 15-month-old APP/PS1 mice, DHA supplementation also decreased Aβ deposition. This suggests that a DHA-enriched diet can diminish AD-like pathology [100]. DHA reduces amyloidogenic β-site cleavage of APP by a direct effect on β-secretase activity and by impairing internalization of BACE1 into the endosomal system [84]. Aβ-releasing γ-secretase activity is also directly affected by DHA. Furthermore, DHA reduces γ-secretase processing of APP in lipid rafts by causing a shift of PS1 (and cholesterol) and thus γ-secretase activity from the raft to non-raft fractions of the membrane. The potential of DHA for the treatment or prevention of AD is further underlined by its cholesterol-lowering effect, which further reduces Aβ generation. DHA reduces cholesterol de novo synthesis by directly inhibiting HMGCR activity, the rate-limiting step in cholesterol de novo synthesis, and by disturbing lipid raft integrity. This directs cholesterol out of these membrane microdomains [84,101,102]. These identified molecular mechanisms result in significant reductions in total Aβ levels and Aβ accumulation in DHA-supplemented cell culture experiments or in animal studies [100,103,104,105,106]. Besides DHA, the n-9 PUFA oleic acid (OA), the most abundant dietary FA, exerts anti-amyloidogenic properties. Transgenic AD mice expressing the Swedish double mutation and Indiana mutation fed a low-fat, cholesterol-free diet enriched with OA exhibited reduced levels of β-site APP cleaving enzyme (BACE) and reduced presenilin levels along with reduced amyloid plaques in the brain [88]. The potency of OA to reduce Aβ levels has been also found in APP695 transfected COS7 cells. Supplementation with OA resulted in reduced secreted Aβ levels [88].

It is discussed that the n-6 PUFA AA, in contrast to the n-3 PUFA DHA, elevates γ-secretase processing of APP, since SP-C99 transfected COS7 cells exposed to AA secreted significantly more Aβ40 and Aβ42 peptides [107]. In cells cultured with 0.1 mM or 0.2 mM linoleic acid (18:2), the precursor of AA, elevated levels of the N-terminal fragment of the γ-secretase component PS1 were found along with increased Aβ levels [108]. In line, an early onset AD transgenic mouse model expressing the Swedish double mutation (K670N/M671L) and Indiana mutation (V717F) revealed higher levels of Aβ and amyloid plaques in brains when mice were fed a diet supplemented with 2% AA [107]. Beside AA, trans fatty acids enhance the amyloidogenic processing of APP leading to elevated Aβ levels in cell culture experiments [92]. Compared with the corresponding cis conformation, trans fatty acids directly affect enzyme activities of β- and γ-secretase. Additionally, trans fatty acids significantly increase *BACE1* gene expression and gene transcription of all γ-secretase components. Notably, the protective effect of DHA is reverted in presence of oxidized DHA. In cell culture experiments, using human neuroblastoma cells and mouse mixed cortical neurons, five different oxidized DHA derivatives and the lipid peroxidation products of n-3 and n-6 PUFAs, HNE and 4-hydroxy-hexenal, revealed elevated Aβ and soluble β-secreted APP levels [86]. The molecular mechanisms leading to higher Aβ levels in presence of oxidized lipids were identified as an increase in gene expression of *BACE1* and the γ-secretase components PS1, Nicastrin and Aph1b, leading to amyloidogenic processing of APP. Additionally, oxidized lipids had a direct stimulating effect on β-secretase activity. Importantly, as little as 1% oxidized DHA was sufficient to reverse the protective effects of DHA and significantly increase Aβ generation [86]. It is therefore necessary, to prevent the oxidation of DHA in dietary approaches or supplements. Supplementation of PUFA must be implemented under conditions that protect from unintentional oxidation.

Due to the anti-oxidative property of vitamin E protecting lipids from oxidation, supplementation with vitamin E is discussed to be beneficial in AD. Additionally, vitamin E has neuroprotective, anti-inflammatory and hypocholesterolemic properties [109]. However, the effect of vitamin E on Aβ generation is still not completely understood and evidence of the beneficial role of vitamin E for the treatment of AD remains inconclusive [110]. While vitamin E supplementation led to reduced cerebral Aβ content and amyloid deposition in young AD transgenic mice, this effect was not observed in aged APP transgenic mice, that received the vitamin E-supplemented diet at an older age [111]. Reduced cerebral levels of Aβ oligomers have been also found in APP/PS1 double transgenic mice fed with α-tocopherol quinine, an oxidative metabolite of α-tocopherol [112]. In contrast, cell culture experiments suggest amyloidogenic potential of different members of the vitamin E family, including α-, γ- and δ-Tocopherol [113]. All tocopherols increased Aβ generation in human neuroblastoma cells by an elevation in gene expression as well as protein level of BACE1 and the components of the γ-secretase complex. In line with the Aβ increasing effect of tocopherols, α-tocotrienol also elevated Aβ levels in human neuroblastoma SH-SY5Y wildtype cells as well as APP695 expressing SH-SY5Y cells. α-Tocotrienol has a direct stimulating effect on the enzyme activities of β- and γ-secretase. Beside these unfavorable properties of members of the vitamin E family with respect to Aβ generation, both tocopherols and α-tocotrienol reduced cholesterol levels, a well-known risk factor for AD [114,115,116]. Due to the partially opposable effects of vitamin E with respect to the molecular mechanisms involved in AD, a recommendation without restriction for the treatment or prevention of AD should be reconsidered. Several epidemiological studies as well as animal studies and cell culture studies show that high cholesterol is linked to elevated Aβ generation and AD pathology [117,118,119,120]. High cholesterol levels have been found to increase β- and γ-secretase activity, especially in cholesterol-rich lipid raft membrane microdomains. In contrast to the E-vitamins, vitamin D has been reported to exert anti-amyloidogenic potential. APP695 transfected SH-SY5Y cells treated with 25(OH) vitamin D3 (calcifediol) or different vitamin D3- and vitamin D2-analogues showed significantly reduced total Aβ levels [90]. Vitamin D3 and its analogues decrease β-secretase activity due to a reduction in *BACE1* gene expression along with a decrease in BACE1 protein level. A 24% reduction in BACE1 protein level was also found in aged rats that received a subcutaneous injection of 1,α25-dihydroxyvitamin D3 (42 I.U./kg for 21 days) [121]. In line with these findings, Aβ40 and Aβ42 levels are elevated in hypovitaminosis D mouse brains caused by an elevated BACE1 protein level resulting in elevated β-secretase activity in these mice [122]. Vitamin D3 and its analogues also decrease gene expression of the γ-secretase component nicastrin, leading to reduced γ-secretase activity [90]. In addition to these molecular mechanisms, another study has found that APP promoter activity is suppressed in presence of 1,α25-dihydroxyvitamin D, indicating that Aβ secretion might be reduced after vitamin D3 treatment due to a diminished gene expression of the Aβ-precursor *APP* [123]. APP transgenic mice fed with a vitamin D3 enriched diet for five months, starting immediately after weaning, also revealed a decrease in the number of amyloid plaques and a reduction in Aβ peptides, further underlining the anti-amyloidogenic potential of vitamin D [124]. However, a recent study reports that vitamin D supplementation worsens the progression of AD [125]. In this study, vitamin D supplementation increased Aβ level and amyloid burden along with elevated BACE1 protein level in the hippocampus of APP/PS1 transgenic mice.

Several cell culture and animal studies dealing with vitamin B supplementation or vitamin B deficiency strongly indicate that B-vitamins exert protective effects with respect to Aβ pathology [126,127,128,129,130]. Tg2576 transgenic mice overexpressing the APP Swedish (APPswe) mutation, resulting in elevated β-secretase processing of APP and therefore Aβ levels, fed with a folate (vitamin B9), vitamin B6 and vitamin B12 deficient diet for 7 months, showed significantly increased levels of Aβ peptides in the hippocampus compared to Tg2576 mice fed with the control diet [126]. In line, elevated Aβ deposition has been reported for TgCRND8 mice (expressing the Indiana mutation in addition to the APPswe mutation), fed with a diet deficient in vitamin B6, vitamin B12 and folate compared to the control diet [127]. In this study elevated Aβ deposition was caused by an increase in *PS1* and *BACE* expression. Besides the identification of alterations in *BACE1* and *PS1* gene expression in presence of vitamin B deficiency, leading to elevated Aβ levels, Aβ levels are increased caused by an elevation in cholesterol de novo synthesis. Human adipocytes cultured in media containing low vitamin B12 or no vitamin B12 revealed significantly increased cholesterol levels caused by an increase in gene expression of genes involved in cholesterol de novo synthesis, including the rate-limiting enzyme *HMGCR* (3-hydroxy-3-methylglutarly CoA reductase) [131] compared to cells cultured with adequate vitamin B12 levels. Furthermore, low vitamin B12 increased gene expression of sterol regulatory element-binding proteins, *SREBP1* and *SREBP2*, and the sterol regulatory element-binding transcription factors, *SREBF1* and *SREBF2*, which are involved in the regulation of cholesterol synthesis and low-density lipoprotein receptor (LDLR) gene expression. Again, hypomethylation of the promoter regions is discussed to play a crucial role in the increase in cholesterol biosynthesis under vitamin B12 deficient conditions [131], leading to elevated Aβ levels. Based on these studies B-vitamins are discussed to reduce amyloid pathology by decreasing *BACE1* and *PS1* gene expression and decreasing cholesterol de novo synthesis [81]. This protective effect of B-vitamins is further underlined by several clinical randomized controlled trials showing the beneficial effects of B-vitamins in persons affected by MCI or AD [81,132,133,134,135].

Among phytochemicals, EGCG is discussed to reduce the amyloidogenic processing of APP by inhibiting β-secretase activity in a cell-free system [136]. In vivo, prolonged administration of EGCG to mice has been shown to down-regulate APP in the hippocampus, suggesting that EGCG might reduce Aβ levels additionally by impairing *APP* gene expression [96]. Resveratrol, derived from a subclass of non-flavonoid polyphenols termed stilbenes (mainly found in red grapes and red wine), is reported to possess anti-amyloidogenic activity, reducing secreted and intracellular Aβ peptide levels in several cell lines expressing the APPswedish mutation [137]. The anti-amyloidogenic activity of resveratrol is discussed to be attributed to an inhibitory effect on the activity of β-secretase as well as by modulating the proteasome [137,138,139]. Furthermore, in vivo studies have demonstrated that resveratrol decreases amyloid plaque formation, Aβ42 levels, BACE1 and APP levels in Tg6799 mice expressing five familial AD mutations (5xFAD mice) [140] and decreases the amount of insoluble Aβ in the hippocampus of AD rats [141]. Genistein, a naturally occurring isoflavone primarily present in legumes, green peas, soybeans, and peanuts, has been found to inhibit BACE1 through reversible non-competitive mechanism, thus reducing Aβ generation [142]. Recently, Genipin, an aglycone isolated from the extract of *Gardenia jasminoides* Ellis fruit, has been reported to decrease Aβ production by inhibiting *BACE1* expression in N2a cells expressing the Swedish mutant of APP [143]. Furthermore, Lepidine B and E from Lepidium sativum have been suggested as potent inhibitors of β-secretase BACE1 in a recent study [144]. Caffeine, that, like DHA, resulted in elevated α-secretase activity, also decreased amyloidogenic APP processing by down-regulating *BACE1* gene expression and directly affecting β-secretase activity [99].

##### Aβ Degradation

Total Aβ level is not only determined by the production of Aβ out of its precursor APP but also strongly depends on its degradation by Aβ-degrading enzymes. An insulin-degrading enzyme (IDE), a zink-metalloprotease, as well as neprilysin (NEP) have been identified as the main enzymes involved in Aβ catabolism [145,146]. Several micronutrients, including PUFAs, DHA, and EPA as well as medium-chain length fatty acids (MCFAs), have been found to enhance Aβ degradation by affecting IDE [147,148] (Figure 1). DHA has been reported to elevate the exosome release of IDE and to directly stimulate IDE activity, leading to an increased Aβ degradation in the extracellular space [147]. EPA also directly stimulates IDE activity, besides elevating gene expression of *IDE*. MCFAs also directly elevate the activity of recombinant IDE and increase extracellular IDE levels, whereas longer-chain length FAs resulted in an inhibited IDE activity [148]. Calcifediol (25(OH) vitamin D3) as well as D3- and D2-analogous have been reported to elevate Aβ degradation in mouse neuroblastoma cells [90]. The increase in Aβ degradation in presence of vitamin D3 or analogous could be mainly attributed to an elevated expression of NEP along with an increase in NEP activity. Calcifediol also elevated the protein level of IDE [122]. In line with an increase in NEP expression and NEP activity in vitamin D-supplemented cell culture experiments, a significant decrease in NEP expression and NEP activity is reported for hypovitaminosis D mouse brains [122]. Vitamin D supplementation (1,α25-dihydroxyvitamin D3, 42 I.U./kg for 21 days) also resulted in significantly elevated NEP protein levels in aged rats [121]. In contrast, members of the vitamin E family, tocopherols as well as tocotrienols, have been found to decrease Aβ degradation by affecting IDE, leading to elevated Aβ levels [149,150].

The monoterpene Geniposide, a major iridoid glycoside of *Gardenia jasminoides*, has been reported to elevate Aβ degradation. The intragastric administration of Geniposide in streptozotocin-induced diabetic rats increased the expression of IDE and decreased Aβ1-42 levels [151]. Similarly, Catalpol increased IDE expression and reduced Aβ levels in mice injected with Aβ and D-galactose [152]. In addition, it has been shown that Catalpol alleviates fibrillar Aβ1-42 induced disruption of the blood–brain barrier and enhances soluble Aβ clearance [153].

##### Aβ Oligomerization, Aβ Aggregation and Aβ Fibrillogenesis

DHA as well as OA were identified as excellent inhibitors of Aβ40 and Aβ42 fibrillogenesis (~81–84% inhibition) in vitro by the use of fluorescence-based aggregation kinetic experiments, transmission electron microscopy and molecular docking studies [154] (Figure 1). EPA, α-linolenic acid (ALA) and AA also exhibit anti-aggregation properties, although to a much lesser extent than DHA and OA [154]. Accordingly, DHA and AA at micellar concentrations stabilized soluble Aβ42 wild-type protofibrils, preventing their conversion to insoluble fibrils [155]. In contrast, fluorescence thioflavin-T-based assay and electron microscopy studies found that trans fatty acids, which already elevate amyloidogenic processing of APP, increase oligomerization and aggregation of Aβ in cell culture studies [92]. B-vitamins, in addition to reducing amyloid pathology by decreasing *PS1* and *BACE1* gene expression and cholesterol de novo synthesis, have promising beneficial properties in relation to Aβ fibrillogenesis and aggregation. Vitamin B12 inhibited Aβ42 aggregation in a dose-dependent manner, significantly prevented the conversion of Aβ42 from random coil to the β-sheet formation and reduced the hydrophobicity of Aβ fibrils as well as the site of the aggregates [81,156]. A recent study further supports the inhibitory effect of vitamin B12 on Aβ fibrillation. Vitamin B12 has been found to significantly reduce Aβ fibril content by reducing the transition of Aβ oligomers to mature fibrils [157]. Furthermore, in presence of synthetic neuronal membranes vitamin B12 has been found to disaggregate preformed fibrils [157]. Besides vitamin B12, vitamin A and provitamin A (β-carotene) inhibit oligomerization of Aβ and destabilize preformed Aβ fibrils [158]. Using fluorescence spectroscopy with Thioflavin T and electron microscopy, it was shown that vitamin A and β-carotene inhibited the formation of fibrillar Aβ from fresh Aβ and its extension in vitro in a dose-dependent manner [159]. In addition, retinoic acid (vitamin A) decreased cellular toxicity by inhibition of Aβ42 oligomerization [160]. In vivo, retinoic acid attenuates Aβ deposition and rescues memory deficits in a transgenic mouse model of AD [161]. A recent study by Joshi et al. further underlines the protective effect of vitamin A with respect to Aβ aggregation in vitro and in vivo [162]. The authors found that vitamin A delayed Aβ42 aggregation in an in vitro screen based on Thioflavin T, whereas α-tocopherol, a vitamin E metabolite, promotes the aggregation of Aβ42. Combining vitamin A and vitamin E has no effect on Aβ42 aggregation. Transmission electron microscopy also revealed a reduction in Aβ42 fibrils in presence of retinoic acid and an increase in Aβ42 fibrils in presence of α-tocopherol. As discussed in the section on Aβ pathology, vitamin E, including α-tocopherol, must be considered an ambiguous player in AD pathology. In animal models, protective effects could be found in relation to Aβ pathology, whereas cell culture studies or in vitro studies revealed potential negative properties [113,162]. However, the study by Joshi et al. further shows that vitamin A, as well as vitamin E, have protective effects in a *Caenorhabditis elegans* model of AD. In this AD model, Aβ42 is expressed in the body wall muscle cells, where it aggregates and results in age-progressive paralysis. Upon treatment with vitamin A and vitamin E, a decrease in Aβ42 aggregates/fibrils along with an increase in the total fitness of the worms compared to untreated worms was detected [162]. Orally administered α-tocopherol also decreased levels of Aβ oligomers in brains of APPswedish/PS1dE9 transgenic mice [112].

Several in vitro and in vivo studies indicate that curcumin, a yellow pigment in the rhizome of turmeric (*Curcuma longa*) has anti-amyloidogenic properties [163]. In vitro studies have demonstrated that curcumin inhibits the formation and extension of neurotoxic Aβ1-40 and Aβ1-42 fibrils from fresh Aβ in a dose-dependent manner and destabilizes preformed fibrils to regenerate Aβ monomers [164,165,166]. Several molecular mechanisms of the anti-amyloidogenic property of curcumin are discussed: (1) curcumin with its two 3,4-methoxyhydroxyphenyl rings connected by a short carbohydrate chain might be able to specifically bind free Aβ and subsequently inhibit the polymerization of Aβ into Aβ fibrils [164]; (2) curcumin might specifically bind to Aβ fibrils and might destabilize the β-sheet-rich conformation of Aβ in Aβ fibrils [164]; (3) curcumin is able to bind to the N-terminus (residues 5–20) of Aβ42 monomers and low molecular weight oligomers [167] and to induce major structural changes in the Aβ1-42 aggregates [168]; (4) curcumin molecules intercalate among the Aβ chains in the first step of Aβ aggregation and bind tightly to them by hydrogen bonds and hydrophobic interactions, leading to less flexible and more disordered amyloid structures [169]. In vivo, systemic treatment with curcumin reduces pre-existing plaques in ~8-month-old APPswe/PS1dE9 mice, suggesting the ability of curcumin to disaggregate Aβ deposits [170]. Besides stimulating α-secretase activity, EGCG interferes with Aβ aggregation. EGCG binds weakly and non-specifically to Aβ monomers, whereas it exhibits higher affinity to oligomers [171]. Electron microscopy revealed that ECGC interferes with the early step of Aβ aggregation by forming spherical, off-pathway aggregates, thus inhibiting secondary nucleation of Aβ [171,172]. Recently, it has been shown, that EGCG is also able to disassemble preformed Aβ fibrils. ECGC has been reported to disrupt Aβ protofibrils by forming π-π and hydrogen bonding interactions [173,174]. Additionally, a recent meta-analysis of 17 studies in AD animal models showed that EGCG can reduce Aβ pathology through anti-aggregating activity in combination with anti-inflammatory and antioxidant properties [175]. The isoflavone genistein also prevents the formation of Aβ aggregates by directly binding to Aβ25-35 fragments [176]. Antifibrillogenic activity has been also reported for *Gingko biloba*. The standardized extract from the leaves of *Gingko biloba* tree, EGb761, prevented β-amyloid fibril formation in solution in vitro as well as in the conditioned medium of neuroblastoma cells stably expressing the Swedish mutant APP and the exon-9 deletion mutant PS1 [177].

#### 2.1.2. Tau Pathology

**Figure 2:**
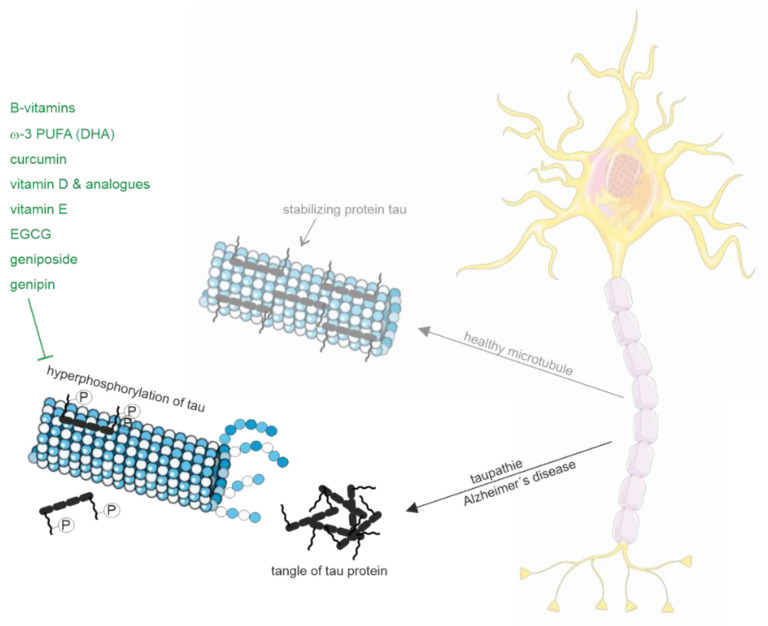
Influence of nutritional components on tau pathology. PUFA: polyunsaturated fatty acid. DHA: docosahexaenoic acid.

Besides the described protective properties of B-vitamins with respect to Aβ pathology, B-vitamins also improve Tau pathology through various molecular mechanisms (Figure 2). Homocysteine-induced changes in Tau hyperphosphorylation could be reduced by simultaneous supplementation of folate and vitamin B12 in rats [129]. In line, supplementation of folate and vitamin B12 alleviates hyperhomocysteinemia-induced Alzheimer-like pathologies, including Tau hyperphosphorylation, in the rat retina [130]. In this context, it has to be mentioned that in the retina, an outgrowth of the developing brain, tau hyperphosphorylation as well as Aβ accumulation are observed in early AD stages [178]. The homocysteine-induced Tau hyperphosphorylation in the hippocampi of rat brains could be ascribed to an inhibition of protein phosphatase 2A (PP2A) involved in Tau dephosphorylation [179]. Tau hyperphosphorylation and PP2A inhibition could be significantly antagonized by the simultaneous supplementation of folate and vitamin B12, indicating that B-vitamins elevate PP2A activity, leading to Tau dephosphorylation [129,179]. Furthermore, B-vitamins are discussed to inhibit several kinases involved in Tau phosphorylation, including glycogen synthase-3β (GSK-3β), cyclin-dependent kinase-5, C-jun N-terminal kinase, extracellular signal-regulated kinase and p38MAPK [129]. In addition, Tau polymerization in presence of vitamin B12 is inhibited by the direct binding of vitamin B12 to cysteine residues of Tau [180]. DHA has also been found to reduce Tau hyperphosphorylation by inhibition of kinases involved in Tau phosphorylation. DHA suppressed traumatic brain injury-induced tau hyperphosphorylation by inhibiting c-jun N-terminal kinases, improving neurological function [181]. The influence of PUFAs on c-jun N-terminal kinase could also be shown by the dietary treatment of 3xTg AD mice [104]. Dietary DHA treatment significantly reduced the steady state level of Tau protein after three months. After six months somatodendritic tau accumulation was significantly reduced in 3xTg AD mice, fed with a diet containing DHA alone or in combination with the n-6 PUFA docosapentaenoic acid (DPA). Mice fed the DHA-DPA diet revealed a reduction in phosphorylated c-Jun N-terminal kinase which correlated with reduced levels of early stage phosphor-tau epitopes. The n-3 PUFA DHA also inhibited c-Jun N-terminal kinase and phosphorylation of Tau in cultured hippocampal neurons and in 3xTg AD mice [182]. Treatment of 3xTg AD mice on a high-fat diet with fish oil or curcumin or a combination of both for 4 months reduced phosphorylated JNK and phosphorylated Tau. Furthermore, DHA-containing phosphatidylcholine showed a reduction in phosphorylated Tau in Aβ25-35-induced AD rats [183]. DHA has also been reported to inhibit GSK-3β phosphorylation and the phosphorylation of Tau proteins in APP/PS1 wildtype mice fed with DHA (400 mg/kg once daily for 2 months), thus inhibiting tau protein neurofibrillary tangle formation in the hippocampi of these mice [184]. Moreover, DHA treatment attenuated increased levels of hippocampal tau phosphorylation in rats fed with a high-fat diet, suggesting that DHA protects against the neurotoxic effects of phosphorylated Tau [185]. Notably, a recent study by Zussy et al. shows that the intranasal administration of nanovectorized DHA decreases the phosphorylation of Tau and restores cognitive functions in two complementary AD murine models, paving the way for the development of new approaches to prevent or treat AD [186].

Besides DHA, recent findings propose beneficial properties of vitamin D or vitamin D analogues with respect to tau pathology. Activation of the vitamin D receptor (VDR) by paricalcitol, a specific agonist of the VDR, reduced phosphorylation of Tau at Ser396 and Thr181 sites via inhibiting GSK-3β phosphorylation in APP/PS1 transgenic mice [187]. Maxacalcitol, an active vitamin D analogue, significantly decreased hyperphosphorylation of MAPK-38, ERK1/2 and tau proteins in experimental AD in rats [188]. In addition to the repressive effect of vitamin D on kinases involved in Tau phosphorylation subcutaneous injection of vitamin D (1,α25-dihydroxyvitamin D3) for 21 days in rats resulted in a 29% increase in PP2A activity in hippocampal tissue [189]. In line with the elevated PP2A activity, Tau phosphorylation in the hippocampus was reduced in aged rats after vitamin D administration, including pre-neurofibrillary tangle phospho-tau protein (pThr231), intraneuronal neurofibrillary tangle phospho-tau protein (pSer214), and extracellular neurofibrillary tangle phospho-tau protein (pSer404). 1,25(OH)2D3 also alleviates Aβ25-35-induced Tau hyperphosphorylation in SH-SY5Y cells [190]. Notably, a recent cross-sectional, explorative study investigating possible associations of vitamin D in CSF with biomarkers for AD, including tau protein and phosphorylated tau protein, revealed that higher levels of 25(OH) vitamin D were significantly associated with lower levels of tau protein as well as phosphorylated tau protein [191].

Vitamin E also exerts protective effects with respect to Tau pathology. Primary cultures of rat cortical neurons incubated with 5 µM β-amyloid peptide cause an oxidative-stress-induced activation of p38 MAPK, leading to tau hyperphosphorylation [192]. The Aβ-induced effects were prevented when neurons were co-incubated with Trolox, the water-soluble analog of vitamin E. Furthermore, high level of phosphorylated p38 MAPK in the hippocampus of APP/PS1 transgenic mice could be prevented by feeding mice with a diet supplemented with vitamin E [192]. Vice versa, dietary deficiency in vitamin E and folate under conditions of oxidative stress increased phospho-tau levels in mice expressing human apolipoprotein E4 (associated with increased risk of AD) [193]. A recent study investigated the effects of vitamin D and E on an insulin-resistant model induced in SK-N-SH neuronal cells, hypothesizing that treatment with vitamin D and E would reverse the effects of AD and improve insulin signaling [194]. Besides the improvement of the insulin signaling pathway upon vitamin D treatment, vitamin D significantly decreased GSK3β and Tau expression levels. Vitamin E alone as well as the combination of vitamin D and E also reduced GSK3β and Tau. In contrast to the proposed beneficial properties of DHA, vitamin B12, vitamin D and vitamin E, vitamin A (retinol) supplementation to human neuroblastoma cells elevated tau phosphorylation at Ser396 [195].

The polyphenol EGCG also exerts potential protective effects in respect to Tau pathology in addition to its protective effects regarding Aβ pathology. EGCG reduces sarkosyl-soluble phosphotau isoforms in TG2576 mice [196]. It is therefore discussed to decrease phospho-tau by direct binding and inhibition of heat shock protein 90 (HSP90), reported to be involved in the phosphorylation status of Tau [197]. Furthermore, Geniposide attenuated Tau hyperphosphorylation by reducing GSK-3 enzyme activity in streptozotocin-treated rats and mice [198,199]. Beside affecting GSK-3 geniposide has been reported to decrease Tau hyperphosphorylation and Aβ42 generation due to increased leptin signaling [200,201]. A recent study further demonstrates the potential beneficial effect of Genipin with respect to tau hyperphosphorylation and Tau fibril formation [143]. In this study, Genipin has been found to bind to Tau and to protect against heparin-induced Tau fibril formation. Additionally, Genipin downregulates the expression of the Tau-kinases CDK5 and GSK-3β in Tau-overexpressing cells.

#### 2.1.3. Oxidative Stress

Oxidative stress, which can be defined as an imbalance between the formation of oxidant species and insufficient antioxidant defense [202], appears to be another hallmark of AD pathology. For example, a study analyzing human postmortem brain samples demonstrated that compared to non-demented control individuals, oxidized lipid and 4-hydroxy-nonenal (HNE) levels were significantly increased in brain samples from PwAD. This negatively affected Aβ levels [86]. Moreover, the levels of hydrogen peroxide, a major reactive oxygen species (ROS), were detected to be increased in the brains of PwAD compared to healthy controls and can be influenced by Aβ peptides [203,204,205]. It is discussed that the overproduction of ROS due to mitochondrial damage occurs earlier than the Aβ pathology or clinical symptoms [206]. Oligodendrocytes are the cells in the central nervous system that may contribute to oxidative stress due to their reduced glutathione levels compared to other brain cells [207]. Oligodendrocytes are the exclusive location for myelin-formation in the central nervous system. Impaired function of this cell type, e.g., due to oxidative stress, can lead to demyelination. This, in turn, reduces the action potential time of neurons and thereby worsens the cognitive decline in PwAD [208].

**Figure 3:**
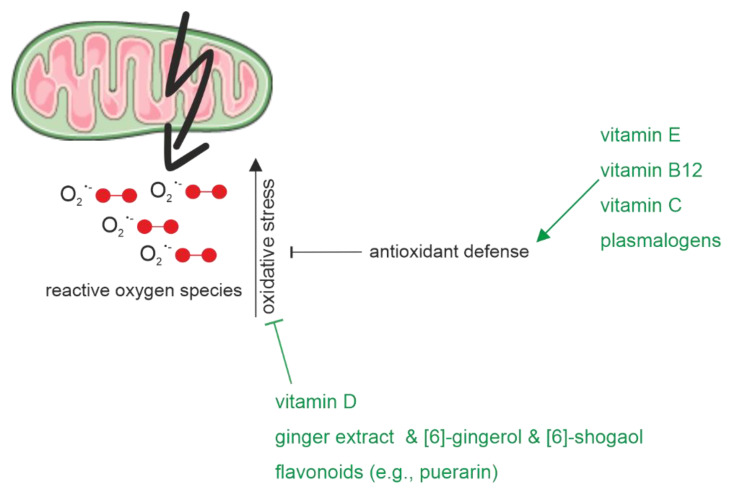
Influence of nutritional components on oxidative stress.

The before-mentioned mechanisms are representative reasons why antioxidants, which prevent and reduce free radical-mediated damage in neuronal cells, are important for the prevention and treatment of AD. Based on this, different pharmacological therapies are currently discussed [209]. In the following paragraph, some antioxidants from food sources and their potential impact on AD pathology due to oxidative stress are presented (Figure 3). One important antioxidant is vitamin E, which might also have beneficial effects on AD pathology due to its neuroprotective properties [210]. Vitamin E occurs in various forms in natural food (four tocopherols and four tocotrienols), of which α-tocopherol represents the most abundant and bioavailable antioxidant in humans [211]. Foods rich in vitamin E are for example vegetable oils, various nuts, seeds, or green leafy vegetables. On a molecular level, vitamin E can be classified as a lipophilic antioxidant that protects membranes from free radical-mediated oxidative damage [212]. The antioxidative properties of vitamin E are based on a hydroxyl group in its phenolic group on the chromanol ring, which can donate a hydrogen atom to neutralize free radicals, including ROS [213]. Besides α-tocopherol, also for α-tocotrienol, the most abundant form of the tocotrienol family, which is characterized by the unsaturated side chain, a reducing influence on the generation of ROS could be detected in human neuroblastoma cells [150]. Furthermore, in an animal study using a transgenic AD model, vitamin E was able to reduce lipid peroxidation [111]. Regarding human clinical or epidemiological studies there are only limited and inconsistent data available analyzing the role of vitamin E alone regarding oxidative stress [210]. Moreover, most of these kinds of studies used a combination of different nutrients or antioxidants, which will be discussed later.

A further fat-soluble vitamin with antioxidative properties is vitamin D. This secosteroid might mediate its beneficial influence on oxidative stress via transcriptional regulation involving the intracellular vitamin D receptor. It is reported that Vitamin D prevents oxidative stress-related oxidation of proteins and lipids as well as DNA damage by the facilitation of balanced mitochondrial activities [214]. On a cellular level, it was shown recently that the active form of vitamin D (1,25(OH)2 vitamin D3) can modulate Aβ-induced ROS by scavenging intracellular ROS [190]. Moreover, similar findings regarding the antioxidant potential of vitamin D were obtained in animal studies [215]. In line with this, increased oxidative stress was reported in an AD mouse model fed with a vitamin D-deficient diet for 13 weeks. Along with this, enzymes such as superoxide dismutase 1 (SOD1), glutathione peroxidase 4 (GPx4) or cystine/glutamate exchanger (xCT) were reported to be downregulated under these conditions [216].

In addition to fat-soluble vitamins, a positive influence on oxidative stress has also been reported for water-soluble vitamins. For example, vitamin B12 was recently shown to protect against the ROS-mediated oxidation of lipids in a cell model. Especially plasmalogens were protected from hydrogen peroxide-induced oxidative stress in the presence of vitamin B12. This was mediated by an increased expression of superoxide-dismutase (SOD) and catalase (CAT), two ROS-degrading enzymes. Furthermore, the transcription of alkylglycerone phosphate synthase (*AGPS*) and choline phosphotransferase 1 (*CHPT1*), two enzymes involved in the plasmalogen synthesis, was also elevated under oxidative stress conditions in the presence of vitamin B12 [217]. Besides vitamin B12, a recent study reported that vitamin C decreased oxidative stress and DNA damage caused by brain surgery (laparotomy) in an APP mouse model [218]. Vitamin C is known to have antioxidant effects [73]. On a structural level, this could be explained by its vulnerability to providing an electron for oxidizing radicals [219]. Moreover, Vitamin C has synergistic effects with vitamin E in the protection of low-density lipoprotein from oxidative damage [220]. In line with this, a study using a mouse model with a knockout of a neuronal vitamin C transporter to generate a vitamin C deficiency reported elevated oxidative stress in the brain cortex and reduced total glutathione in comparison to wild-type mice [221]. Moreover, supplementation of vitamin C (3.3 g/l) was able to prevent abnormal mitochondrial morphology found in vitamin C deficient 5x FAD mice [222]. Along with these findings in animal studies, a meta-analysis found a lowered relative risk (0.83 with 95% CI 0.72 to 0.94) for AD when vitamin C was consumed in the diet [223].

Regarding plasmalogens, it should be mentioned that this lipid class has an impact on the generation and caused damage to oxidative stress itself. This is due to characteristic moieties in their unique chemical structure: plasmalogens have a vinyl-ether bond at the sn-1 position of the glycerol-backbone, which makes them highly susceptible to oxidation. Furthermore, they have bound PUFAs at the sn-2 position frequently, which are also vulnerable to oxidative stress [224]. Analysis of cerebral cortex homogenates of rats under induced oxidative stress confirmed that plasmalogens are highly sensitive to oxidative stress. This was demonstrated, for example, by the 70% decrease in plasmalogens after 90 min of UV irradiation [225]. These and other findings suggest plasmalogens as antioxidant molecules, as the vinyl ether bond could be the first target for newly formed radicals.

Moreover, the beneficial effects of ginger, which has numerous medical properties, and its biologically active components including, among others, gingerols, shogaols, paradols, and zingerone, regarding AD were reported. It was reported in vitro, for example, that ginger extracts were able to prevent lipid peroxidation in rat brains [226]. In neuroblastoma cells, pretreatment with [227]-gingerol prevented the Aβ25-35-induced disruption of the mitochondrial membrane potential and effectively inhibited the accumulation of ROS by restoring endogenous antioxidant glutathione levels and upregulating the expression of antioxidative enzymes [228]. Moreover, also for [6]-shogaol it could be shown, that this component of ginger could recover an increased ROS production induced by H_2_O_2_ treatment in vitro [229].

Another nutritional factor with anti-oxidative properties is flavonoids. For example, for the natural isoflavone puerarin, an alleviation in oxidative stress was reported in an AD mouse model [230]. It seems to be unlikely that flavonoids represent direct ROS scavengers due to their low circulating concentrations in the brain [231]. More likely is the modulation of pathways, that include for example pro-survival signaling molecules such as Akt/protein kinase B, p38 mitogen-activated protein kinase or c-jun N-terminal kinase [232]. An additional mechanism of action was described in vitro and comprises the activation of transcription factors, such as Nrf2 or PPARγ [233].

Oxidative stress can, among others, lead to inflammation by the activation of microglia and astrocytes, which results in the release of pro-inflammatory cytokines. Inflammation, along with Aβ- and tau-pathology is a further hallmark of multifactorial AD and may also contribute to and exacerbate this neurodegenerative disorder. Due to this, the following paragraph is going to present the influence of nutritional components on inflammatory processes related to AD.

#### 2.1.4. Inflammation

A further contributor to the development and exacerbation of AD is inflammation, which is mediated by astrocytes and microglia in the brain. Microglia are considered the major source of pro-inflammatory cytokines such as IL-6, IL-1β or TNF-α. These cytokines are important for the regulation and initiation of the inflammation process including the migration of leukocytes and immune cells.

**Figure 4:**
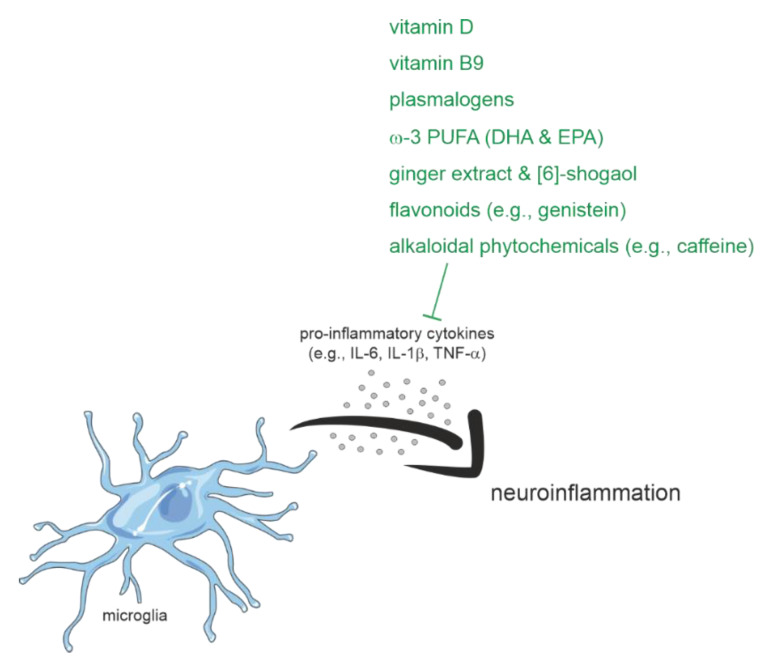
Influence of nutritional components on inflammation. PUFA: polyunsaturated fatty acid. DHA: docosahexaenoic acid. EPA: eicosapentaenoic acid.

In the study mentioned above, it was found in the mouse model that, due to vitamin D malnutrition, increased inflammatory stress in form of promoted glial activation and significantly increased secretion of inflammatory factors (IL-1β, IL-6, and TNFα) developed [216] (Figure 4). Another recent study, using vitamin D-deficient mouse brains for genomics analysis, reported a significantly increased expression of *Casp4*, a gene encoding caspase-4, which is part of the innate immune response [234]. Microglial caspase-4 expression is suggested to contribute to cognitive impairments in AD, such as hippocampal synaptic plasticity [235]. On a molecular level, it could be shown in human neuroblastoma cells that a supplementation of vitamin D and its analogues is able to tendentially decrease the levels of IL-1β [90]. Moreover, for acitretin, a derivate of the fat-soluble vitamin A, which is known to mediate anti-inflammatory properties [236], an immune stimulatory effect was shown in the 5xFAD mouse model and human CSF [237].

With respect to water-soluble vitamins, a randomized controlled trial reported decreased inflammation due to the supplementation of folic acid (vitamin B9). In a study including 120 people, a six-month treatment with 1.25 mg folic acid per day resulted in significantly lowered levels of *TNF-α* mRNA in participants of the intervention group compared to those of the control group. Based on these findings, the authors suggested inflammation as an essential player in the association between folic acid and AD [238].

Similar findings were obtained for plasmalogens on the cellular level recently. Pretreatment of human neuroblastoma cells with eicosapentaenoic acid-enriched ethanolamine plasmalogen in a neuroinflammation model, generated with help of conditioned medium from lipopolysaccharides-induced BV2 cells, resulted in the reversal of increased nitric oxide and TNF-α levels as well as of the reduced IL-10 levels [239]. Regarding lipids and fatty acids, a study found new evidence for the molecular mechanisms through which ω-3 fatty acids, EPA and DHA, may exert their known anti-inflammatory and neuroprotective properties [240]. A pre-treatment of human hippocampal progenitor cells with EPA or DHA prevented the decrease in neurogenesis and the increase in apoptosis, which both were induced by treatment with IL-1β, IL-6 and interferon-α (IFN-α) [241]. Moreover, a further study indicates another possible underlying mechanism since ω-3 PUFAs were found to attenuate the inflammation-induced hyperactivity of the immunoproteasomes in astrocytes [242]. In a rat model, the supplementation of EPA was able to normalize the IL-1β-induced elevation in *TNF-α* expression and thereby mediate beneficial effects regarding inflammatory processes [243].

Additionally, for some bioactive components of ginger beneficial effects regarding neuroinflammation were reported. For example, it could be shown that [6]-shogaol exerts anti-inflammatory influences by inhibiting the microglia-mediated production of proinflammatory cytokines (IL-1β, TNF-α) in vitro [244]. Similar findings were obtained in the brain of animal models of dementia, where treatment with [6]-shogaol was reported to play a role in inhibiting glial cell activation [245]. Moreover, ginger extract can inhibit Aβ-induced expression of proinflammatory genes, including TNF-α, COX-2, or IL-1β in a human monocytic cell line sharing properties with human microglial cells [246].

Regarding the numerous beneficial effects of flavonoids on neuroinflammation [247], it was for example recently reported, that genistein, an isoflavone abundant in soy, mediates its inhibitory effects on LPS-induced expression of TNF-α, IL-1β or IL-6 via the activation of a G protein-coupled estrogen receptor (GPER) [248]. Animal studies found the natural flavonoid eriodictyol, to regulate inflammatory mediators and cytokines via the NF-κb and MAPK pathways [249].

In the context of potential neuroprotective agents, phytochemicals should not be disregarded as they have, for example, beneficial effects on the neuroinflammatory cascade. With regard to AD pathology, alkaloidal phytochemicals, such as caffeine, berberine, huperzine A, galantamine, sophocarpidine, or nicotine, are of interest, among others, because of their neuroinflammatory properties, as recently reviewed [250]. Besides these nutritional approaches, the gut microbiota has become a new potential target to alleviate neuroinflammatory processes in AD, in recent years [251].

#### 2.1.5. Multicomponent Nutritional Interventions

Regarding nutritional interventions, more and more evidence arises that combined dietary components show synergistic beneficial actions with respect to AD pathology. For example, it could be shown in an AD mouse model that the dietary vitamin E status influences the benefits of fish oil supplementation. With respect to oxidative stress, a dose-dependent attenuation was observed, expressed as modulatory effects on the antioxidant system [252]. A recent in vitro study found a preventive effect of the Fortasyn Connect multi-nutrient combination on reactive astrogliosis. This combination of DHA, EPA, uridine monophosphate, choline, phospholipids, folic acid, vitamins B12, B6, C, and E, and selenium, was able to prevent the reactive astrogliosis typical molecular and morphological changes that were induced by pro-inflammatory cytokines TNF-α and IFN-γ [253]. Moreover, in another study, various transcriptional patterns were detected in blood samples of participants having a diet supplemented with olives (olive oil), nuts (MUFA, PUFA, fibers, vitamin E), or fish (ω-3 fatty acids) according to the Mediterranean diet. Compared to controls, several genes were found to be differentially expressed and those were associated with inflammation, as for example *IL-8*, *STK17B*, or *RGS1* [254]. In summary, the current literature suggests a combination of vitamins (vitamin D, vitamin B complex), fatty acids (the ω-3 fatty acids DHA and EPA), flavonoids (e.g., resveratrol), alkaloids (e.g., caffeine), and polyphenols (e.g., curcuminoids) as nutritional supplements for PwAD [255].

Based on the before presented findings regarding the beneficial potential of some nutritional components in the context of AD, clinical studies were performed, using the daily medical food Souvenaid (Nutricia Advanced Medical Nutrition), which contains a combination of DHA, EPA, phospholipids, choline, uridine monophosphate, folic acid, selenium, and the vitamins B12, B6, C, and E (Fortasyn Connect) [256]. For example, using APPswe/PS1DE9 transgenic mice, it has been demonstrated that diet can be considered a modifiable risk factor for prodromal and early AD. A 3-week intervention of a diet with Fortasyn was able to elevate markers of cholinergic synapses and to improve muscarinic neurotransmission in this transgenic AD mouse model [257]. Based on these promising findings, the first non-pharmacological intervention study, the LipiDiDiet trial, was performed. This double-blind and multicenter RCT was designed to analyze the influence of Fortasyn Connect on cognitive performance in prodromal PwAD. The readout was assessed using a neuropsychological test battery [258]. In 2021, the results of the 36-month intervention from 81 (36 control and 45 active) participants at the prodromal stage of AD from eleven study sites in Sweden, Finland, the Netherlands, and Germany were published. They reported significant reductions in the decline in cognition, memory, brain atrophy and AD progression. In summary, these findings indicate that the duration of the intervention as well as their starting point regarding the disease stage are crucial for their success [259].

Indications for the beneficial role of the combined intervention strategies regarding the progression of AD are also given by animal studies. For example, a recent study using a rat model, in which AD was induced by administering 70 mg/kg aluminum chloride via intraperitoneal injection for five weeks, reported that natural antioxidants enhance the effect of mental and physical activities. This was evident from the fact that the weekly expose to physical and mental activities combined with a treatment of 10 mg/kg EGCG (intraperitoneal injection), 400 mg/kg Vitamin C (per os), 100 mg/kg Vitamin E (per mouth) and 1 mg/kg selenium (per os) elevated the levels of total antioxidant capacity, superoxide dismutase (SOD), brain monoamines as well as the brain-derived neurotrophic factor (BDNF). Moreover, Aβ-, tau- or β-secretase levels were found to be reduced under these intervention conditions [260].

### 2.2. Physical (in)Activity and Alzheimer’s Disease

The extent, volume and intensity of physical (in) activity are closely related to health or illness, well-being and quality of life.

Physical inactivity has become one of the leading health risk factors globally, long underestimated, ranking among the frontrunners both in terms of attributable contribution to total deaths and DALY burden (DALY: disability-adjusted life years) [261]. Physical inactivity leads to a significant reduction in life expectancy and quality of life, as well as a marked increase in the likelihood of occurrence of many non-communicable diseases such as cardiovascular disease, type 2 diabetes mellitus and cancer [262,263]. Among the risk factors linked to dementia or AD, lack of physical activity plays a key role in increasing the prevalence of the disease [264].

In order to counteract the negative consequences of an inactive lifestyle, targeted promotion of physical activity is therefore of great importance for the physical health of PwAD, moreover, it has a positive effect on mental health and cognitive function [26]. Understanding the mechanisms behind the beneficial effects of physical activity and how physical activity can exert neuroprotective actions on the central nervous system are goals that have not yet been achieved. Rody et al. (2022) reiterate that the disease must be treated in the early stages, before symptoms appear, and that the combination of multiple healthy lifestyle factors can be a promising strategy. The same authors warn that physical activity in patients with cognitive decline or AD is a challenge and becomes more challenging when mobility problems are associated. In this case, the adherence and engagement of these individuals to long-term treatments can be, for example, maximized with virtual reality-based physical activity with exergames. However, more studies are needed to indicate the benefits of this type of treatment.

A meta-analysis by Aarsland et al. (2010), which included a total of 24 longitudinal studies involving 1378 people with vascular dementia, showed a significantly reduced risk of 0.62 (95% CI 0.42 to 0.92) for developing vascular dementia in physically active individuals [227].

Further studies have also found a preventive effect of regular physical activity in relation to the development of AD (summarized by Pedersen and Saltin, 2015 [265]). These findings are indirectly supported by a study by Nyberg et al. (2014), which found that low cardiovascular fitness was associated with an up to a 7-fold increased risk (HR 7.34, 95% CI 5.08 to 10.58) for early onset dementia. The greatest risks for early onset dementia and cognitive impairment were found in individuals with low cardiovascular fitness and low cognitive performance [266].

Several studies also investigated the effectiveness of regular physical activity in people with dementia or AD. A Cochrane review by Forbes et al. (2015) found evidence of the benefit of physical activity in improving cognitive performance in people with dementia [267]. The mean difference between the intervention and control groups was 0.43 (95% CI −0.05 to 0.92, *p* = 0.08). Positive significant effects of physical activity were found in relation to the performance of activities of daily living (ADL) in people with dementia. The predictable standardized mean difference between the control and intervention groups was 0.68 (95% CI 0.08 to 1.27, *p*-value 0.02). Furthermore, a reduction in burden was found for caregivers monitoring the participation of a family member with dementia in a physical activity program. The mean difference between the control and intervention groups was −15.30 (95% CI −24.73 to −5.87; *p* = 0.001).

A study by Kemoun et al. (2010) found an improvement in cognitive function in older people with dementia who participated in physical training for 15 weeks [268]. In contrast, participants in the control group experienced a decline in cognitive function. A similar result was seen regarding walking ability (walking speed and step length), which also improved in the intervention group, while it deteriorated in the control group. Positive effects of physical activity in relation to physical functions in older people with dementia were also found by Rolland et al. (2007) [269] and Steinberg et al. (2009) [270].

Furthermore, there is evidence that physical activity also has a positive effect on cognitive function in people with cognitive impairment but without dementia [271,272]. Erickson et al. (2011) also found a positive effect of physical activity on hippocampal volume [273].

Overall, it can be stated that there is good evidence for the effectiveness of the physical activity, especially regarding the prevention of dementia. The same applies to the effects of physical activity in people with dementia in relation to physical functions such as walking ability [265]. Physical activity might also have positive effects on the general function of PwAD [274].

Physical activity appears to have a direct positive impact on brain structures as well as indirectly reducing the risk of dementia via improved cardiometabolic functions. Another major advantage of physical activity is that virtually no risks or side effects have been identified and that individually adapted physical activities can be performed safely and with low risk [265,275].

The homeostasis of mitochondrial function appears to be linked to neural plasticity and the effects of physical activity. Sun et al. (2022) summarized the evidence in a review study where they point out that preventive physical activity for psychiatric and neurodegenerative disorders is efficient due to its effect on mitochondrial and neurogenic functions [276]. The effects of physical activity on neural and metabolic properties are not yet fully understood, but there is strong evidence that physical activity might decrease the inflammatory process, strength the neurogenesis and induce neuroprotective effects [276,277].

Against this background, physical activity also ranks highly in the World Health Organization (WHO) recommendations for dementia prevention [278] and is strongly recommended. Besides physical activity, there is only a strong recommendation for tobacco cessation. Conditional recommendations include a Mediterranean-like diet, interventions to treat alcohol use disorders, cognitive training, weight management, management of hypertension, management of diabetes and management of dyslipidemia (Table 1). Interdisciplinary cooperation is essential for progress in this field. As evidence, Chen et al. (2022) found that most publications that study physical activity and AD involve neuroscience, geriatrics, sports sciences, psychology, and rehabilitation [279].

In fact, several authors confirm that there is no sufficient evidence regarding the different types of physical activity, the different intensities, durations and frequencies, or the related mechanisms [26,279]. Nevertheless, different types of physical activities are recommended, including single strength and endurance training, but also multidomain training programs [280,281,282,283]. Endurance training might be particularly effective and produces greater effects than strength training. However, the optimal dose or specific dose–response relationships are still poorly understood and thus not well defined or derivable. Good randomized controlled trials should be conducted to define these parameters and assess the effects at different stages of the disease.

According to the current activity recommendations of the WHO (2020) for adults (aged 18–64 years) and for older adults (65 years and older), suggesting moderate-intensity aerobic physical activity for at least 150–300 min or 75–150 min of vigorous-intensity aerobic physical activity or a comparable combination of moderate- and vigorous-intensity activity throughout the week, would be necessary to bring substantial health benefits. Further, they recommend that muscle-strengthening activities should be practiced at moderate or greater intensity, including all major muscle groups on two or more days a week. In order to enhance functional capacity and prevent falls in older adults (65 years and older), it is recommended to incorporate in the lifestyle a diverse multicomponent physical activity on three or more days a week that emphasizes functional balance and strength training at moderate or greater intensity [284]. A recent review suggests that combined muscle strength, balance and motor function training can improve postural stability in older adults with AD and reduce cases of falls [285]. However, this type of training does not seem to be different for elderly people without AD. More studies should be carried out to identify whether these individuals have different physiological and biomechanical dysfunctions related to falls compared to healthy elderly people.

Although many steps have been taken in this field, there are still many open questions regarding physical activity and AD. Multidisciplinary work should be encouraged and treatment and prevention strategies should be designed considering the multidomain aspect of the disease.

### 2.3. Cognition-Oriented Treatments

COT are aimed at improving and/or maintaining cognitive processes of daily life functions to warrant participation as long as possible [7,22,286]. COT comprise three components, differing in the addressed focus of intervention: cognitive training, cognitive stimulation, and cognitive rehabilitation. While cognitive training (CT) is directed at restoring specific cognitive abilities such as memory, attention or problem solving through repeated practice of standardized tasks [7,25,286], cognitive stimulation (CST) can be regarded as a psychosocial intervention, mostly organized in group sessions [24,286,287]. Cognitive skills (language, memory, executive functions) are implicitly addressed in nonspecific tasks to trigger executive functions, language, and social interaction [7,24,287,288,289,290]. Cognitive rehabilitation (CR) is a person-centered approach, focusing on individual needs and goals to manage everyday activities and is mostly offered as an individual intervention. Characteristically, people with dementia are involved in goal setting and support for carers is offered [1,2,7,24,25,291,292]. The underlying mechanisms of effective COT are ascribed to the use of cognitive reserve, enabling people with AD to maintain functions despite evident disease pathology [2,293,294,295,296,297]. Results of an functional magnetic resonance imaging study confirmed that despite a significant reduction in grey matter volume CST led in comparison to usual treatment to an increase in resting-state default mode network connectivity in the posterior cingulate cortex and bilateral parietal cortices in PwAD [295]. 

Thus far, numerous reviews on the topic of COT with varying focus but promising results to maintain cognitive health in dementia were published [1,7,25,286,290,298]. In a recently issued overview of 46 systematic reviews on the effectiveness of COT in older adults on cognitive and non-cognitive outcomes, 23 reviews referred to MCI and the dementia population [25]. Overall, CT was associated with small mean effect size estimates on cognition in MCI (*n* = 5; Hedges’ g = 0.40) and dementia (*n* = 7; 0.38). No general statement could be made about the effectiveness of a computerized CT, compared to active or inactive controls, on cognitive function in adults with MCI [293]. In terms of psychosocial outcomes, there were heterogeneous results for MCI and no benefits for dementia. CST in dementia led to small mean effect size estimates on cognition (*n* = 5; 0.32) and on psychosocial outcomes (*n* = 4; 0.26). Compared to CT and CST, there are only few studies that focus on CR since achieving personalized daily living goals is difficult to assess in RCTs. Nevertheless, some trials exist. They demonstrated, for example, significant improvements in participants’ and informants’ ratings of attaining personally relevant everyday goals in early stage dementia [299] as well as reduced functional decline and delay in institutionalization [300]. Since there is an urgent need for approaches targeting daily functioning and its assessment, CR has recently gained increasing attention [2,299,301].

All in all, the included studies exhibited high heterogeneity in reported results and low to moderate methodological quality. Thus far, functional ability and caregiver outcomes have been poorly addressed [25].

#### 2.3.1. Speech and Language Therapy within the Scope of Cognition-Oriented Treatments

Communication as a function of cognitive processes is already affected in the early stages of AD disease. In disease progression, reduced communication can form a major burden for PwAD and caregivers [302,303]. Therefore, it is astonishing that there is a lack of studies on communication-based interventions in dementia [297,304]. Language and/or communication functions are rarely assessed as specific outcome parameters [25,288,290,305,306,307] or rather represent higher cognitive functions as fluency tasks [306,308]. Table 2 provides an overview of cognitive language and communication difficulties and resources as AD progresses.

#### 2.3.2. Effects on Language and Communication after Cognitive Stimulation

The systematic review by Lobbia et al. [290] showed moderate evidence for CST on language production and comprehension, communication skills and quality of life for people with mild to moderate AD, partly even with long-term-effects in studies published after this review [305,306]. These beneficial effects can possibly be attributed to the focus of CST on language activities and communicative interaction between group participants [290,309]. Even with moderate-severe AD communication-based group settings, independent of their format (e.g., CST, conversation therapy, reminiscence therapy [310,311] or treatments that combine social interaction with procedural tasks as described in the “breakfast club” [312] seem to maintain functional communication skills and improve language impairments [297].

#### 2.3.3. Effects on Language and Communication after Cognitive Training and Cognitive Rehabilitation

Language therapy-specific research mostly includes studies of CT and CR approaches or a mix of both. More recently, a person-centered approach with individually determined goals has become prevalent in speech and language therapy to maximize communication success and quality of life [297,311,313]. Thus far, language therapy research is rarely considered in reviews dealing with COT, mostly due to small sample sizes and low evidence levels [24,286,290,304]. However, unlike RCTs, single-case research designs allow the evaluation of personalized daily living goals, which are a central component of CR [23,314]. A review of interventions focused on language and communication outcomes for people with AD [304] described, that lexical-semantic training approaches showed the greatest evidence for naming and word fluency tasks with mixed effects in terms of maintenance [315,316,317]. Furthermore, techniques such as spaced retrieval principles, memory-based approaches and errorless learning [304,318,319,320,321,322,323] are used to reduce the cognitive load in the lexical retrieval process. However, studies also indicate that especially in the early stage of AD errorless and trial-and-error tasks benefit comparably [2,299,324,325]. Beales et al. [326] see potential in strategy-based approaches, to achieve generalizations in lexical retrieval and connected speech. They achieved significant improvements in naming trained and untrained items in four participants with AD, directly after intervention and in a 6-week follow-up assessment. No change was found in communicative informativeness and efficiency. Nevertheless, the authors suggest that the chosen outcome parameters should be critically considered in order to successfully evaluate communication functions [326].

#### 2.3.4. Dyadic Intervention Approaches and Communication Success

Studies emphasize communication partner engagement in dementia interventions as a potentially relevant factor in communication success and confidence [299,303,313,327,328]. Nguyen et al. (2019) demonstrated in a review and meta-analysis that caregiver support intervention groups, addressing educational and psychosocial needs, skills and communication training [299,320,329], achieved significant improvements in daily interactions in dementia care compared with control groups (95% CI 0.56 to 1.22; *p* < 0.001) [303]. Improved communication skills could even be maintained months after interventions (95% CI 0.67 to 1.05; *p* < 0.001). However, heterogeneity between studies was evident [303].

#### 2.3.5. Non-Pharmacological Interventions and Functional Outcomes

To date, evidence regarding functional outcomes/ADL is sparse. According to a systematic review by Scott et al. [301] on non-pharmacological interventions to reduce the functional decline in PwAD living at home, only individually delivered interventions such as CR and tailored physical activity had an effect on functional abilities. These interventions ideally involve the PwAD and their caregivers, whereby structured guidance is critical to achieve personally relevant goals [299].

**Table 2:** Characteristics of cognitive language and communication difficulties in Alzheimer’s disease [330,331,332].

	Language and Communication		Cognition	
	Impairments	Resources	Impairments	Resources
**Mild Stage**	**Reception** - Comprehension of abstract language/complex conversation	- Comprehension of simple sentences - Reading comprehension	- Declarative/explicit memory - Inconsistent problems with orientation - Visuospatial skills - Divided/selective attention - Inconsistent problems with instrumental activities of daily living (IADL)	- Nondeclarative/sensory memory - Awareness of language and memory lapses - Sustained attention - Concentration
	**Production** - Word retrieval for names, objects, locations - Semantic paraphasias - Irrelevant/vague comments - Reduced content/error repairs in discourse	- Grammatical correct sentences - Phonology/articulation - Oral reading/writing		
**Moderate Stage**	**Reception** - Comprehending complexinstructions/tasks - Reading comprehension	- Reading comprehension for familiar words/phrases	- Declarative memory - Orientation - Executive functions - Attention in all domains - Visuospatial skills	- Nondeclarative/sensory memory
	**Production** - Word retrieval - Increase in circumlocutions/word repetition/paraphasias - Disrupted conversation flow - Decline in sentence length/ grammatical complexity/propositional content - Increase in the use of pronouns/vague terms - Lack of content in conversation - Pragmatic abilities: maintain topics of conversation/knowledge of conversation perspectives/irrelevant content/inaccurate utterances	- Phonology - Syntax - Oral reading of simple texts - Nonverbal conversation		
**Severe Stage**	**Reception** - Auditory and reading comprehension	- Comprehension/interpretation of emotional state via facial expression/gestures/eye contact/prosody/voice tone	- Memory - Attention - Fluctuated alertness	- Affective response to sensory stimuli/music - Basic needs for attention/communication/touch present
	**Production** - Production of single words/short phrases - Often inappropriate verbal/vocal production - Repetitive vocal/physical behavior - Mutism in the end stage	- Communication via facial expressions/gestures/eye contact		

### 2.4. Oral Health and Alzheimer’s Disease

There is rising evidence for an association between intraoral inflammatory conditions and AD [17,18,19]. More than 50% of all people over the age of 35 worldwide are affected by periodontitis, and approximately 10–15% suffer from a severe form of the disease which can lead to the loss of dentition [333]. In particular, people over the age of 65 are at increased risk for periodontitis.

Periodontitis is a chronic, bacterial, host-dependent inflammation of tooth-supporting tissues that ultimately leads to loss of the periodontal attachment and, in continuum, to tooth loss through the activation of host proteinases [334,335]. While gingival inflammation that precedes periodontitis is generally triggered by the presence of biofilm, the development and progression of periodontal inflammation depend on the presence of oral dysbiosis [336]. Local degradation products and inflammation-induced milieu changes provide a selection advantage to specific periodontal pathogens such as the Gram-negative anaerobic species known as the “red complex” [337]. These pathogens include *Porphyromonas gingivalis* (*P. gingivalis*), *Tannerella forsythia* (*T. forsysthia*), and *Treponema denticola* (*T. denticola*). Additional risk factors, such as smoking, can exacerbate dysbiotic changes [338,339]. 

In this process, the dysbiotic biofilm causes not only a local inflammatory response but also a systemic inflammatory response, which is related to various noncommunicable diseases and can influence their development and progression [340]. 

Such a relationship is also discussed for dementia, especially AD, and the intraoral condition, which may be bidirectional: on the one hand, the reduction in memory capacity caused by AD is certainly causal for the decrease in personal oral hygiene ability and thus ultimately for the increase in the risk for the development of caries and periodontitis [341,342]. On the other hand, there is evidence that periodontitis itself and the associated microbial colonization and inflammatory response is associated with AD [18,19,343,344].

While the systematic reviews that included cross-sectional studies failed to assume a causal relationship between the presence of periodontal disease and the development of AD [344,345], a more recent systematic review that included only longitudinal data from observational studies came to the conclusion that causality was evident and that, considering the adjusted pooled risk ratio for dementia related to the periodontal disease of 1.38 (1.01 to 1.90), a fictive 75% reduction in the prevalence of severe periodontitis would have the potential to prevent far more than 1 million people worldwide from developing dementia such as AD [346].

Thus far, the underlying mechanisms in the involvement of periodontitis in AD have not been conclusively elucidated. Systemic inflammation, as described above, is one approach. Among others, increased IL-1, TNF-α, and IL-6 plasma levels play an important role, resulting in an increase in microglial activation [347]. The involvement of oral microorganisms and their metabolites is another frequently discussed aspect in the development and progression of dementia. The presence of the bacterium *T. denticola* associated with periodontitis was discovered in brain biopsies from deceased PwAD as early as 20 years ago [348]. The increased presence of antibodies against periodontal pathogens in the blood of persons suffering from dementia also suggested that these pathogens may play an important role in the etiopathogenesis of cognitive diseases [349,350,351]. The bacterium *P. gingivalis* plays a key role in this context because of its virulence factors [352]. This representative of the “red complex” was also recently shown to be increased in brain biopsies from PwAD [353]. *P. gingivalis* uses membrane lipopolysaccharides and special proteases, so-called gingipains, to bypass host defenses and degrade brain tissue. Gingipains in particular are thought to be highly relevant with regard to the initiation of the AD process and have therefore also led to the development of a new drug therapy approach—a gingipain inhibitor [353]. However, such a compound (COR388), which was later clinically tested in a phase II/III trial, failed to lead to therapeutic success and was discontinued—also due to side effects [354,355].

Even though the mechanism of involvement or even causality of periodontitis in AD has not yet been fully clarified, it is obvious to pay attention to early periodontitis diagnosis and its prevention or therapy in the attempt at AD prevention. Nevertheless, there are only few reliable clinical data available to date that deal with a possible improvement of AD through a therapy of periodontal diseases. Regarding the impact of dental and periodontal treatment on cognitive function before and after dental treatment, a longitudinal observational study has previously found that oral restoration of PwAD has the potential to improve cognitive function [356]. Another longitudinal study also demonstrated that regular oral home care had a positive effect on cognitive status [357]. For this purpose, current guidelines recommend brushing with a manual or electric toothbrush twice a day for at least two minutes, in combination with interdental care using interdental brushes [358]. In addition, the application of fluoridated toothpaste and mouth rinses based on Chlorhexamed or essential oils is effective [359]. As dementia progresses, this default can usually not be achieved by the persons themselves at some point, so professional support in-home care becomes necessary to maintain intraoral health as long as possible [360]. Recent data show that a dental nurse who professionally brushed the teeth of nursing home residents every 2 weeks after an initial professional dental cleaning in addition to daily care by relatives or caregivers could contribute to the reduction in intraoral inflammation and minimization of oral problems, such as root caries or tooth loss [361].

In summary, given the potential links between periodontal inflammation and AD, minimizing intraoral biofilm, and consequently reducing the inflammatory response is most likely to be beneficial with respect to the development and progression of dementia. For other chronic diseases, such as cardiovascular disease, this benefit has already been adequately demonstrated: For example, a recent systematic review with meta-analysis concluded that professional and home-based management of oral biofilm as a non-pharmacological intervention reduced systemic inflammation to an extent equivalent to that achieved by drug interventions to treat residual cardiovascular risk [340]. 

Thus, the goal should be to assess intraoral status soon after AD diagnosis and, if necessary, to remove any initial supra- and subgingival plaque by mechanical debridement with or without adjunctive use of antiseptics or antibiotics, followed by long-term individualized professional follow-up in combination with instructions on home oral hygiene for the person himself or a family or professional caregiver, optionally supported by a dental nurse to ensure the longest possible maintenance of oral health [362,363].

**Figure 5:**
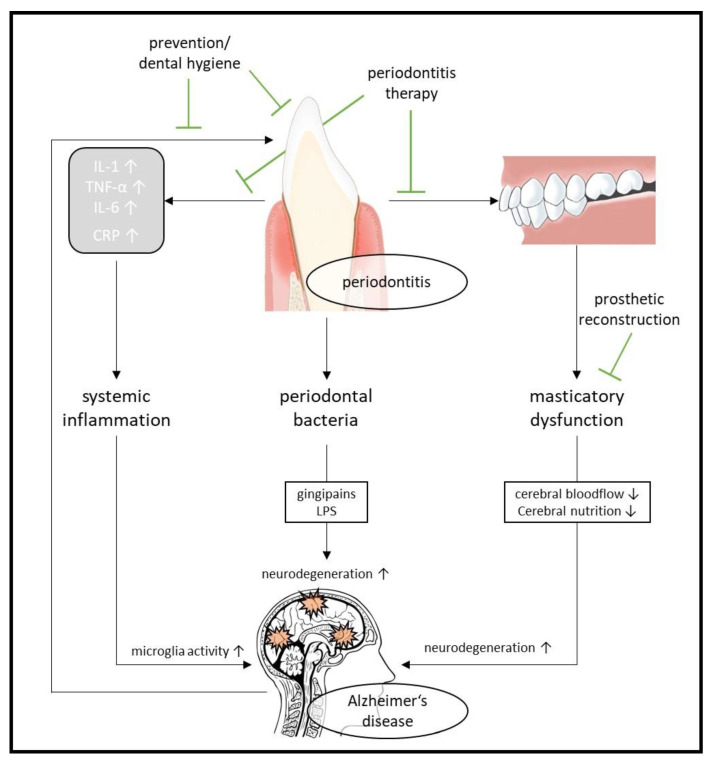
Schematic (simplified) diagram of currently discussed relationships between oral status and Alzheimer’s disease and possible dental interventions.

In addition to the possible mechanisms described above in the association of periodontitis and AD, tooth loss itself, which may result from periodontitis, also plays an important role as a risk factor for the development and progression of dementia (Figure 5). Reduced masticatory function due to tooth loss affects cerebral blood flow and nutrition [364] and led to a decrease in acetylcholine levels and the number of pyramidal cells in the hippocampus when studied in animals, which was associated with a disturbance in learning memory [365]. A recent systematic review, reporting on further animal studies, shows that the main mechanisms in the association of tooth loss and AD may be accelerated neurodegeneration caused by the loss of masticatory function and the accompanying decreased nerve stimulation, as well as a chronic systemic inflammatory stress state [366].

A further recent systematic review of case–control studies shows, in addition to aspects of the systemic effects of underlying diseases such as periodontitis, that tooth loss itself has an impact on the prognosis of dementia and is therefore a fundamental starting point in the planning of dental therapy for elderly persons: The goal should be, if possible, to maintain masticatory function through long-term functional tooth preservation, as this not only has a positive effect on cerebral blood flow and the consequent oxygenation of the brain but also allows for a favorable dementia-protective diet [367]. One possible therapy could be the provision of the missing support zones resulting from tooth loss by means of prosthetic replacement. It has been shown that the swallowing disorders and dysphagia associated with tooth loss could be improved by the use of prostheses, which had an effect not only on the nutritional status but also on the ability to perform ADL [368]. Further improvement of masticatory function was achieved by the additional support of such prostheses on implants placed in the jawbone, resulting in the need for intensive professional follow-up to protect the patients from possible future peri-implant infection [369]. Improvement of mastication by the provision of prosthesis and the therapy of oral complaints led to an increase in the social activity of patients [370]. The provision of dentures was also shown to be beneficial in terms of cognitive function [371].

The influence of individual oral processes and conditions on the oral health-related quality of life of people with AD has been demonstrated recently [372] as well as the long-known assumption that intraoral problems lead to pain and functional limitations resulting in a disease-promoting deterioration of physical, psychological, and social condition [373].

The ideal control of problems associated with tooth loss and oral diseases is the maintenance of oral health and the prevention of tooth loss long before the risk of developing dementia increases. It has already been successfully demonstrated that almost complete tooth preservation can be achieved through a lifelong, individualized and needs-oriented prevention program that begins in adolescence and includes regular check-ups, dental cleanings and instructions on oral hygiene provided by dental hygienists [374].

Although the current evidence suggests a bidirectional relationship between oral status and AD, there are so far only initial clinical studies on the effect of dental treatment of AD status. These are usually observational studies with small numbers of subjects, but they show promising initial results. Currently, initial intervention studies are in preparation, which already considers a first multidomain approach.

## 3. Multidomain Interventions

**Figure 6:**
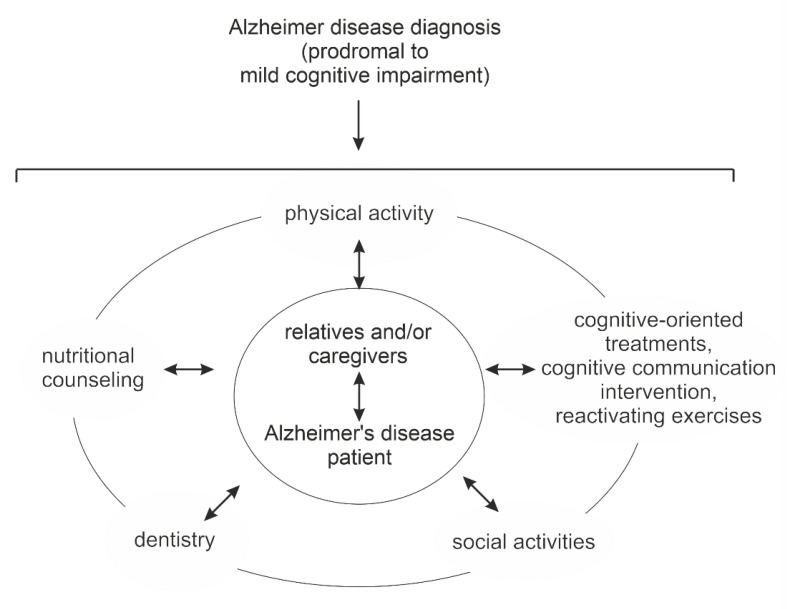
Schematic overview of the interrelationships and interactions of individual interventions in Alzheimer’s disease.

According to the BPS model [20], dementia is a complex condition, that involves interrelated tractable and fixed risk factors. Acting on several modifiable risk factors simultaneously seems to be particularly promising to influence the brain and cognitive reserve and maintain functioning [16,375,376]. Therefore, multidomain approaches, including psychosocial and educational support are of growing interest to delay the decline in dementia [16,314,377,378,379,380]. Figure 6 provides an overview of the interrelationships and interactions of individual interventions in AD.

Two recent reviews on multidomain interventions are of particular interest. The meta-analysis of Salzmann et al. (2022) on 28 studies directly compared multi- and single-domain interventions in MCI. The authors found greater pooled effect sizes in multidomain intervention groups for MCI compared to single-domain intervention groups in global cognition (SMD, 0.41; 95% CI 0.23 to 0.59; *p* < 0.001), executive function (SMD, 0.20; 95% CI 0.04 to 0.36; *p* = 0.01), memory (SMD, 0.29; 95% CI 0.14 to 0.45; *p* < 0.001) and verbal fluency (SMD, 0.30; 95% CI 0.12 to 0.49; *p* = 0.001) [379]. The systematic review of Chalfont et al. (2021) on multidomain interventions in dementia included 26 studies, of which 92% demonstrated improvement, maintenance, or delayed decline of cognitive functioning. Both reviews comprised, among other modes, cognitive, physical, and nutritional interventions, and educational support. Therefore, it is reasonable to combine these approaches, whereby cognition and physical training is up to date the most common combination [314,379,381,382]. A review of 17 studies found greater improvement in older adults with MCI on global cognition (SMD = 0.83, 95% CI 0.41 to 1.25; *p* = 0.0001) for combined cognitive and physical intervention compared with intervention in a single domain [381].

However, according to the review of Chalfont et al. (2020), to improve cognition, at least three modalities should be combined and one of them should have a cognitive approach. Moreover, the greatest effect sizes were identified in studies with individually tailored multidomain approaches [314]. Thus, interventions should be adapted to the individual’s needs and preferences to maintain motivation for active participation in therapy [314,383,384] and to reduce functional decline [301]. Caregiver involvement also seems to be crucial in multidomain interventions [314], especially when it comprises targeted caregiver components [385]. Astonishingly, the severity and length of intervention were not determinants of treatment in the review of Chalfont et al. (2020). However, continuous treatment could be crucial for maintaining functional abilities [386] or physical health [383] in PwAD. The multidomain activation therapy study Motor Stimulation, Activities of daily living, and Cognitive and Social Functioning (MAKS) led to stable results in ADL and cognition of persons with moderate dementia during a 12-month intervention period, whereas an increase in impairment for both measures was found in the control group. In a 10-month follow-up test scores of both groups deteriorated significantly, but scores in ADL remained significantly higher in the intervention group compared to the control group [386]. Prick et al. [383] considered too low therapy intensity crucial for missing therapy effects in mood, behavior and physical health after a multidomain intervention.

As indicated in the before mentioned studies, multidomain lifestyle intervention strategies could be beneficial regarding AD pathology and have therefore been the subject of different clinical trials [387]. A recent RCT with 67 participants over 60 years without any cognitive dysfunction reported improved cognitive functions after combined aerobic and memory training and the supplementation of the functional food soy peptide in form of a commercial drink for 90 days [388].

With reference to study participants, three large prevention trials were performed in the past years, namely preDIVA, MAPT, and FINGER. The preDIVA (Prevention of Dementia by Intensive Vascular Care) study was a cluster-randomized controlled trial in more than 3400 community-dwelling older individuals (aged 70–78 years), examining the effectiveness of a six-year multidomain vascular care intervention to prevent dementia. The intervention included the assessment of cardiovascular risk factors every four months and individually tailored lifestyle advice. No differences in the outcome cumulative incidence of dementia or incident cardiovascular disease between the intervention (more than 1890 participants) and control group (1636 participants), which obtained usual care, was found. One possible reason for the obtained results is seen in the high standards of usual care and a modest baseline cardiovascular risk. However, importantly, as a secondary outcome in the intervention group, a significantly reduced risk of non-AD dementia could be detected [389]. 

The Multidomain Alzheimer Preventive Trial (MAPT) included more than 1500 participants and was conducted over three years. In a randomized, placebo-controlled design the intervention took place in more than 40 group sessions with the content of nutrition, COT, physical activity as well as three preventive consultations. As a specific compound, some of the non-demented community-dwelling participants received ω-3 PUFAs. No significant differences were found in cognition between the intervention groups. However, in comparison to the placebo group, there was a significantly less cognitive decline in the multidomain + PUFA group [390].

The randomized controlled Finnish Geriatric Intervention Study to Prevent Cognitive Impairment and Disability (FINGER) ran for two years and included more than 1200 at-risk elderly people. Interventions were conducted in group and individual sessions comprising diet, COT, physical exercise, and vascular risk monitoring. The control group received usual health care. Results revealed a significant between-group difference in the change in cognition [378] and were not modified by parameters such as cognition, socioeconomic status, cardiovascular factors, or sociodemographic status [391]. Thus, the findings underline the potential benefit of FINGER in a large elderly population to maintain or improve cognitive functioning [378,391]. Based on these results, the current step is to expand the FINGER-type trials to a World-Wide FINGERS (WW-FINGERS) network, facilitating not only international collaborations but also the opportunity to elaborate globally implementable and effective preventive approaches [392,393]. 

Multidomain lifestyle interventions are a current research topic. Among others, the Multimodal Preventive Trial for Alzheimer’s Disease (MIND-ADmini) also examines the potential effects of multidomain interventions in individuals with prodromal AD. Up to date more than 90 participants were randomized and enrolled for the multidomain lifestyle intervention alone or in combination with medical food (Fortasyn Connect) in comparison to regular health advice/care in the control group [394].

The ongoing randomized controlled Japan-Multimodal Intervention Trial (J-MINT) uses original outcome parameters as cognitive changes at 18-month follow-up, changes in ADL, frailty status, blood markers, dementia-related blood bio-makers, and neuroimaging to verify whether the multidomain intervention could prevent the progression of cognitive decline among older adults with MCI [395]. The intervention consists of the management of vascular risk factors, group-based physical activity and self-monitoring of physical activity, nutritional counseling, and COT. 

The first approach of interdisciplinary cooperation of dental hygiene and physiotherapy can be found in the planned study of Jockusch et al. (2022) aiming to activate the masticatory function to improve cognition in AD [396].

Comparable to single-domain interventions, communication-based interventions and outcomes have rarely been considered in multidomain approaches so far. A combination of stimulative walking activity and conversation did not result in a significant decrease in nonredundant information in connected speech compared to a structured conversation-only group [384], among others probably because of limited mobility in the combined group, which may have led to an overload of cognitive capacity [384]. The Language-Enriched Exercise Plus Socialization (LEEPS) Program combined language stimulation with physical activity in older adults with AD and related disorders, leading to stability in cognition, mood, and physical performance over 11 months and in a small group even over 20 months. However, there was a high dropout rate, mainly due to entry into long-term care, worsening health or death [377]. While language abilities were not specifically tested in the study of La Rue et al. (2015), Arkin and Mahendra (2001) used communication-based outcome measures in a similar approach. Although experimental and control groups did not differ in the Arizona Battery for Communication Disorders of Dementia (ABCD) [397], the specific analysis showed a significantly more differentiated use of nouns in the experimental group. However, results were based on case series [398].

## 4. Discussion

A large-scale meta-analysis and systematic review of more than 150 RCTs and more than 240 observational prospective studies identified evidence-based modifiable factors for the prevention of AD [399]. The Lancet Commission also recommends addressing risk factors that favor the development of dementia across the lifespan, as this can affect cognitive reserve and delay neuropathological developments [16,314]. Physical activity and cognitive activities stimulate cerebral blood flow and the production of neurotrophic factors that influence hippocampal neuroplasticity [314,382,400]. In particular, physical activity and dietary interventions are known to address similar mechanisms involved in AD. For example, a reduction in oxidative stress or effects on mitochondria, in general, are known to be involved in both disciplines [401]. In this context, the effect of PGC-1α, a mitochondrial super-regulator, has been reported to be affected by AD and might be an interesting target for further studies [402]. Moreover, dietary interventions influence cellular energy metabolism and can therefore have an impact on synaptic plasticity [314], a parameter, which highly correlates with cognitive reserve and cognitive activity in general. Synaptic plasticity is further influenced by different lipids [403]. E.g., plasmalogens or phosphatidyl-choline in combination with choline/UMP-choline or DHA have been shown or are at least discussed to increase several synaptic markers such as synaptophysin or PSD95 [404,405]. Interventions in the prodromal and early stages of AD may correct the deficiency of key nutritional elements that could otherwise lead to the loss of synapses and neurons [256]. 

Obviously, both physical activity and dietary interventions elevate cerebral blood flow, another important factor necessary for cognitive activity. These are only a few examples that combine different approaches to adjust the dysregulated metabolic homeostasis in AD synergistically from different angles, resulting in an elevated beneficial potential with respect to AD compared to approaches based on a single intervention. A further important aspect is the multifactorial component of AD: as reviewed above, AD can be caused by several different molecular mechanisms. Apparently, addressing only one mechanism has been proven to be not sufficient to deal with AD up to now, making a multidisciplinary approach, addressing these different risk factors or molecular mechanisms involved in AD, even more attractive. 

In line with this argumentation, multidomain interventions have already been shown to be effective in stimulating modifiable risk factors in parallel to delay the progression of dementia [314,379,382], even with existing structural changes in the brain [295,400,406]. To improve cognition, multidomain interventions should comprise at least three modalities, one of which is a cognitive approach [314]. Up to now, cognition and physical training is the most common combination of the multidomain approaches [314,379,381,382] whereby nutritional interventions and educational support play an important role in supporting and counseling PwAD and their families throughout the course of the disease [378,385,407,408]. The success of psychoeducational interventions depends on clear communication of theoretical contents and the active involvement of carers in practice sequences to implement new skills [303,385]. Support of oral hygiene and health, prevention and diagnosis of periodontal disease are promising approaches in dementia care that have not yet been addressed in multidomain interventions but should receive more attention in the future [363,367,371,409]. In this context, diabetes mellitus, recently identified as an additional risk factor for the development of AD [410], should also be mentioned, even though the underlying mechanisms have not yet been clarified [411]. It is known that there is a bidirectional relationship between the presence of intraoral inflammation, especially periodontitis, and diabetes mellitus [412]. Thus, an improvement of the intraoral condition also fulfills the previously formulated need to implement a diabetes mellitus-protective lifestyle for AD prevention [399].

Person-centered approaches are a characteristic feature of CR [23,292,299] and similarly, multidomain interventions with individualized contents achieve the greatest effect sizes [314]. 

Regarding nutritional interventions, more and more evidence arises that combined dietary components show synergistic beneficial actions with respect to AD pathology [255,256]. Multidomain interventions that target preventive, or early stage disease show potential for delaying the onset of dementia [259,378,389,390]. Dietary modifications combined with aerobic physical activity also led to cognitive improvements in participants with elevated blood pressure [413]. Since high blood pressure is also a risk factor for the onset and progression of AD, combined nutritional approaches with specific physical activity are promising for AD. 

In many studies, cognition is the primary outcome used to test the effectiveness of the intervention. Unfortunately, most of the chosen assessments or outcomes have little relation to the demands of daily living [314,326], but rather measure higher cognitive functions [308]. This is all the more astonishing because impairments in communication already occur in the early phase of the disease. Future work should therefore consider communication skills as an outcome measure. Corresponding parameters would be, for example, the proportion of topic-related utterances and empty utterances or the global coherence in the discourse of people with dementia [288,305].

Although there is a growing interest in the effects of dental therapy on the occurrence and progression of AD, dentistry still has a minor role in the multidisciplinary context. Despite interrelated biochemical processes in intraoral inflammation and AD [349,355], there are few clinical studies investigating the effectiveness of oral treatment approaches in AD. Difficulties in recruitment include ethical issues, the health status of people with disabilities, lack of compliance, required involvement of relatives, treatment limitations, and economic issues [351,396,414]. However, oral treatment approaches are very promising to be integrated into a multidomain approach. In particular, approaches to nutrition, oral hygiene, mobility and masticatory function can be optimally coordinated and adapted to individual needs, which overall contributes to an improvement in the quality of life of PwAD. Sessions can take place in groups as well as individually and thus promote social activities, as is also recommended in this context [16].

Future research should address the feasibility of multidomain approaches in clinical practice. To establish this approach, orientation sheets can be developed and evaluated. A proposal for an interdisciplinary approach to nutrition therapy/counseling is outlined below (Table 3).

## 5. Conclusions

**Figure 7:**
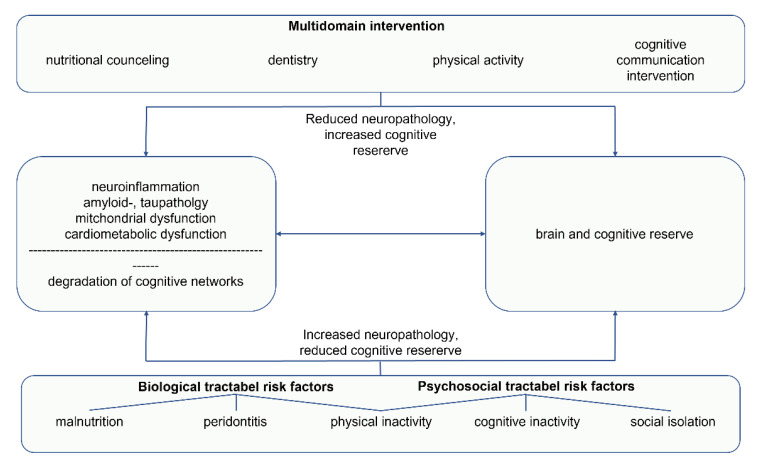
Association between biological and psychosocial tractable risk factors, intervention, neuropathology, and cognitive reserve.

Pleiotropic mechanisms are known to result in AD. Although these molecular mechanisms are well-known and can be addressed by pharmaceutical and non-pharmaceutical approaches, including nutrition, physical activity or increasing the cognitive reserve, beneficial effects for PwAD are not sufficient to cope with the disease. However, treatments based on multicomponent or even multidisciplinary approaches revealed more pronounced benefits. Unfortunately, the number of studies and the different included disciplines are limited. For example, oral health, which obviously interferes with nutritional status but also many other factors reviewed above, is mostly neglected. Therefore, we suggest a tight interlink between different treatment strategies resulting in an interdisciplinary approach, covering in particular nutritional counseling, supervised physical training, oral health, and cognitive-oriented communication training to maintain quality of life in AD (Figure 7). Importantly, all of these single approaches are already available now and the required interlink between these approaches seems to be feasible and associated with only a moderate additional effort. As an example, a simple route card for PwAD or nutritional counselors, caregivers, or relatives addressing these interlinks is suggested, helping to individualize and adopt treatment. Due to the synergistic action of these interventions, a significantly greater benefit for the PwAD might be expected. Even before the onset of AD, the approaches listed could potentially be considered preventive approaches. The awareness of the presented correlations and the possible preventive effectiveness should also be discussed with patients who do not yet suffer from AD. Although the reviewed literature is promising, further studies addressing these interdisciplinary aims are needed to prove effectiveness.

## Figures and Tables

**Table 1:** WHO recommendations for risk reduction in cognitive decline and dementia (source: own illustration according to World Health Organization, 2019).

WHO Recommendations	
Strong recommendation	- Physical activity (aerobic, resistance training or multicomponent physical activity) - Tobacco cessation
Conditional recommendation	- Mediterranean-like diet - Interventions to treat alcohol use disorders - Cognitive training - Weight management - Management of hypertension - Management of diabetes - Management of dyslipidaemia

**Table 3:** Nutritional Intervention: A suggested 6-step program for nutritional counselors.

6-Step Program		
Step	Involved Disciplines	Content Clarification and Coordination
1	Oral health/dentist	- Potential risk factors that interfere with food intake? - Dental status sufficient that food intake is not perceived as unpleasant? - Xerostomia, dysphagia present? - Problems present in the mechanical comminution of food? - Current antibiotic treatment due to periodontitis etc.?
2	Speech and language therapy	- Clarification of existing dysphagia/dysphagia intervention - Language/reading comprehension present? - What to consider when communicating? - Participation in social/communication groups?
3	Sport science and physiotherapy	- Age corrected grip strength test to estimate malnutrition available? - WHO recommended sports load possible? (How many times/duration per week) - Sport practice preferably under sunlight? - Participation in regular sport activity groups? - Short individual sport program for home available?
4	Medical performance	- Signs of malnutrition present based on blood test (albumin, vitamin D, selene, vitamin B12)? - Any antibiotic treatments? (e.g., antibiotics and calcium; vitamin K and anticoagulant drugs) - Any medication that interferes with drug-degrading enzymes? (such as Cyp) - Any redocumentations that interfere with vitamin uptake or uptake of essential fatty acid? (proton-pump inhibitors)
5	Relatives/professional caregivers	- Any food intolerances or personal preferences such as vegetarian diets? - Regular food intake helps to structure the daily routine - Feeding or food intake should be done in an upright position and not lying down to avoid aspiration pneumonias - Utilize food intake, cooking classes, cooking in general to prevent social isolation - Use (of liked) foods to create sensory stimuli - Participation in social groups? E.g., regular meetings for a joint breakfast?
6	Nutritional advice	- Adjust caloric intake to achieve an age-appropriate normal weight - Avoid unintentional weight loss - When dental health affects food intake, additive use of liquid food might be necessary - Explain the Mediterranean diet: rich in antioxidants, vitamins, PUFA, polyphenols, phytochemicals, and vegetables in general - Explain potential beneficial use of medium chain fatty acids (coconut oil) - Supplementation of vitamin B12, vitamin D - Consider LipiDiDiet-based supplementation - Ensure sufficient intake of omega 3 fatty acids such as DHA (e.g., two times/week fatty sea fish) - Avoid trans fatty acid and highly processed food in general - In case of antibiotic treatment: make sure that the microbiome is built up afterwards through nutrition (increase fiber intake, probiotics if necessary) - Consider further phytochemicals as supplementation such as EGCG (or green tea extract), Ginseng, Gingko, resveratrol, etc. - Moderate coffee consumption

## Data Availability

Not applicable.
